# Assessing vegetation recovery from energy development using a dynamic reference approach

**DOI:** 10.1002/ece3.8508

**Published:** 2022-02-17

**Authors:** Adrian P. Monroe, Travis W. Nauman, Cameron L. Aldridge, Michael S. O’Donnell, Michael C. Duniway, Brian S. Cade, Daniel J. Manier, Patrick J. Anderson

**Affiliations:** ^1^ U.S. Geological Survey Fort Collins Science Center Fort Collins Colorado USA; ^2^ Natural Resource Ecology Laboratory Colorado State University, in cooperation with the U.S. Geological Survey, Fort Collins Science Center Fort Collins Colorado USA; ^3^ U.S. Geological Survey Southwest Biological Science Center Moab Utah USA

**Keywords:** *Artemisia*, quantile regression, resilience, sagebrush, soil properties, weather

## Abstract

Ecologically relevant references are useful for evaluating ecosystem recovery, but references that are temporally static may be less useful when environmental conditions and disturbances are spatially and temporally heterogeneous. This challenge is particularly acute for ecosystems dominated by sagebrush (*Artemisia* spp.), where communities may require decades to recover from disturbance. We demonstrated application of a dynamic reference approach to studying sagebrush recovery using three decades of sagebrush cover estimates from remote sensing (1985–2018). We modelled recovery on former oil and gas well pads (*n* = 1200) across southwestern Wyoming, USA, relative to paired references identified by the Disturbance Automated Reference Toolset. We also used quantile regression to account for unmodelled heterogeneity in recovery, and projected recovery from similar disturbance across the landscape. Responses to weather and site‐level factors often differed among quantiles, and sagebrush recovery on former well pads increased more when paired reference sites had greater sagebrush cover. Little (<5%) of the landscape was projected to recover within 100 years for low to mid quantiles, and recovery often occurred at higher elevations with cool and moist annual conditions. Conversely, 48%–78% of the landscape recovered quickly (within 25 years) for high quantiles of sagebrush cover. Our study demonstrates advantages of using dynamic reference sites when studying vegetation recovery, as well as how additional inferences obtained from quantile regression can inform management.

## Graphical abstract

Using remote sensing and ecologically relevant reference locations, we applied a dynamic reference approach to studying sagebrush cover following energy development. Quantile regression indicated little of the landscape would recover under poor to median restoration conditions (low to mid quantiles), but also identified areas unlikely to recovery even under optimistic restoration conditions (high quantiles).

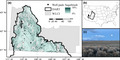

## 1 INTRODUCTION

The global extent and magnitude of anthropogenic disturbance are unprecedented, and widespread preservation and restoration efforts are needed to avert or reverse losses in biodiversity and ecosystem function (Wolff et al., 2018). Restoring ecosystems through different methods and across scales provides many promising benefits to mitigate environmental degradation (Perring et al., 2015). However, challenges remain in identifying appropriate restoration targets in dynamic landscapes and when working at large extents. To calibrate restoration strategies and evaluate outcomes, reference sites are needed to represent the expected or potential state of an ecosystem at recovery (Aronson et al., 1995; Brinson & Rheinhardt, 1996; Herrick et al., 2019; Society for Ecological Restoration International Science & Policy Working Group, 2004). A reference condition is identified spatially, such as via ecological site potentials (Herrick et al., 2019; Nauman & Duniway, 2016), but defining its relevant timeframe is not trivial (White & Walker, 1997). Indeed, environmental contexts and disturbance vary over space and time in frequency, magnitude, and scale, and conditions that produced a historical reference may no longer exist for contemporary restoration (Jackson & Hobbs, 2009; Kirkman et al., 2013; Seastedt et al., 2008). Relevance of a temporally static reference to recovering systems can therefore be ambiguous (Hiers et al., 2012; Hobbs, 2007; Thorpe & Stanley, 2011; White & Walker, 1997).

The temporal conundrum of identifying reference conditions may be resolved by defining a reference that can change over time (hereafter, dynamic reference), whereby restoration objectives are more likely to resemble current and future states of the reference (Choi, 2004; Hobbs & Norton, 1996; Pickett & Parker, 1994). Previous applications of the dynamic reference concept used time‐varying measurements of community composition and distance‐based ordination to evaluate the state and trajectory of restoration (Hiers et al., 2012; Kirkman et al., 2013). While these studies determined targets for restoration in a changing environment, identifying mechanisms behind variation in recovery is necessary when making predictions over space and time (Brudvig, 2017). Examining recovery relative to dynamic reference sites, for example, may indicate factors that accelerate recovery, or reveal when and why deviations from a desired trajectory occur, thereby signaling the need for additional interventions. Furthermore, a dynamic reference approach can reduce uncertainty when assessing restoration treatments by accounting for factors such as climatic variation and sensor noise in remote sensing studies (Fick et al., 2021). Such an approach also could reveal restoration constraints including abiotic conditions, legacy factors, and landscape context (Aronson & Le Floc'h, 1996; Suding, 2011).

Across western North America, semi‐arid ecosystems are often dominated by sagebrush (*Artemisia* spp.), a keystone species important for a variety of wildlife species including greater sage‐grouse (*Centrocercus urophasianus*; Fedy et al., 2014). In these environments, sagebrush communities may require over a century to recover from disturbance (Avirmed et al., 2015; Baker, 2006; Lesica et al., 2007), and in the intervening recovery time, temperature and moisture availability naturally fluctuate but also are projected to change across the sagebrush range (Kleinhesselink & Adler, 2018; Renwick et al., 2017; Schlaepfer et al., 2012a). Interannual variability and long‐term trends in climate indicate that conditions during restoration and recovery will likely differ from historic references. Therefore, we argue the importance of using dynamic references and accounting for mechanisms that underly variation in order to monitor and evaluate post‐disturbance outcomes effectively.

Restoration efforts generally require monitoring and intervention to increase success, such as additional treatment or maintenance (Tischew et al., 2010), and multiple decades of monitoring sagebrush ecosystems may be required to produce conclusive results. To further complicate matters, sagebrush ecosystems, and restoration efforts within, extend across a vast and variable landscape. Together, these temporal and spatial considerations highlight the need for alternatives to long‐term, ground‐based monitoring for broad‐scale inferences, such as using remote sensing and archived data to track change over time (Kennedy et al., 2014; Shi et al., 2018; Xian et al., 2015). For example, recovery rates on former oil and gas well pads were estimated across a study landscape while considering factors such as weather and soils (Monroe et al., 2020); however, this study did not consider dynamic reference conditions and instead evaluated recovery relative to a static, pre‐disturbance condition. By restricting analyses to well pads with pre‐disturbance data within the timeframe of historical remote sensing imagery (<30 years), inferences were substantially constrained for a system that may require >70 years to recover (Avirmed et al., 2015). Concomitantly, Nauman and Duniway (2016) developed the Disturbance Automated Reference Toolset (DART) to identify areas with equivalent ecological potential near disturbed sites based on a suite of environmental attributes. This latter approach was then used to compare reference and disturbed sites (Nauman et al., 2017). While DART indicated relative differences in recovery at a fixed point in time, the assumption of equivalence between space and time is often uncertain and may obscure mechanisms behind trends in recovery (Pickett, 1989). Further work with DART looked at time series analysis of differences in a soil adjusted total vegetation index (SATVI) at oil and gas well pads and selected reference areas to understand timing of recovery better, but the generalized nature of SATVI was often confounded by annual invasive species when interpreting recovery trends (Waller et al., 2018).

Here, we demonstrated a dynamic reference approach to studying sagebrush recovery following energy development in southwestern Wyoming, USA, by applying DART to back‐in‐time remote sensing products specifically representing sagebrush cover. First, we used DART to identify ecologically relevant reference areas near former oil and gas well pad areas (hereafter, well pads), and second, we estimated annual sagebrush cover in both disturbed and reference areas over three decades. We then modelled annual sagebrush cover on disturbed sites relative to paired, dynamic references while considering multiple environmental factors, thereby circumventing assumptions of space‐for‐time. Finally, we used these models to project time to recovery across the study landscape. Sagebrush recovery depends on various local, landscape, and historical factors (Pyke, 2011), only a fraction of which can be reliably quantified and included as covariates in models. Disturbance also may push sites into alternative states that cannot recover without additional interventions (Hobbs, 2007; Pyke, 2011), and therefore may show different responses to environmental factors than sites in other ecological states. A novel aspect of the approach described here is our use of quantile regression (Koenker & Bassett, 1978) to model recovery with dynamic references. In addition to dispensing with typical assumptions of linear regression that may be untenable in complex ecological systems, such as specific error distributions and homogeneity of variance, quantile regression is useful for modelling trends that are likely to be influenced by limiting factors and unmodelled variation (Cade & Noon, 2003; Shinneman & McIlroy, 2016).

## 2 STUDY AREA

**FIGURE 1:**
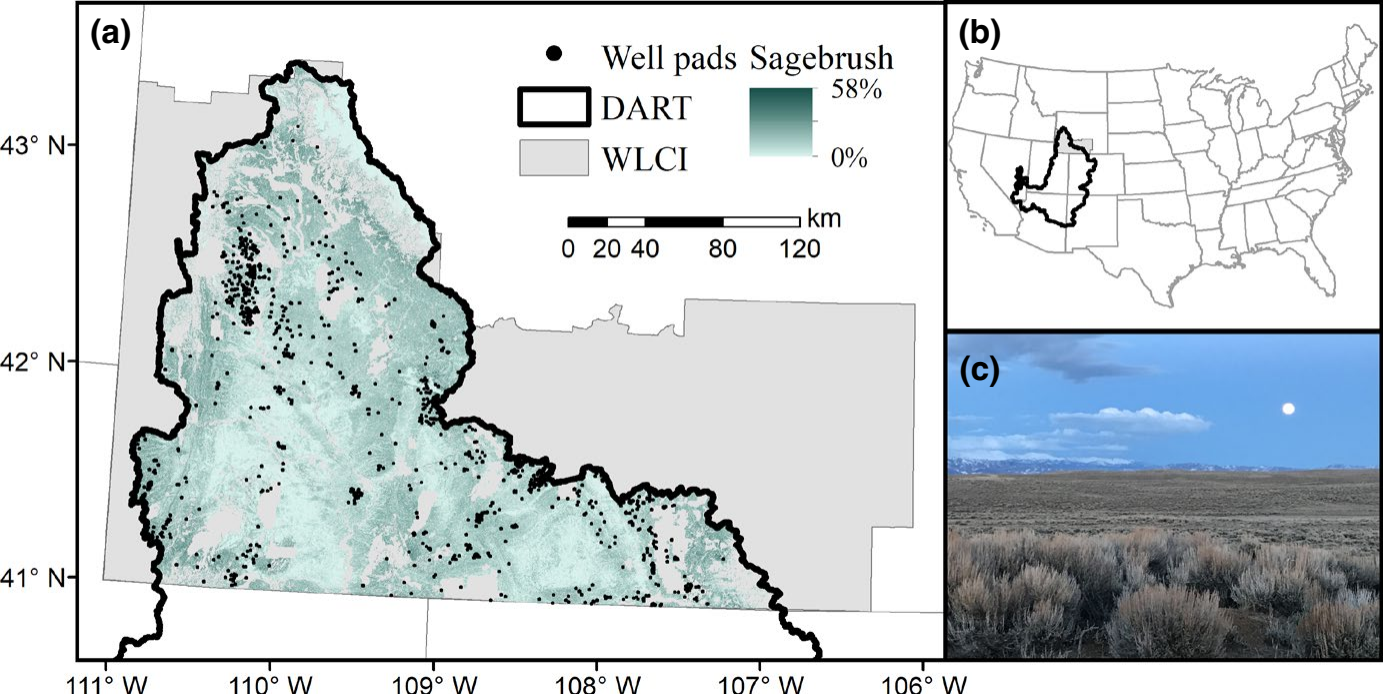
Locations of former oil and gas well pads (*n* = 1200; a) in southwestern Wyoming, USA (b). The study area overlaps the Wyoming Landscape Conservation Initiative area and the extent of the Disturbance Automated Reference Toolset developed for the Upper Colorado River Basin. We also present percent cover (a) of sagebrush (*Artemisia* spp.; c) summarized by 95th percentile across the data time series (1985–2018, excluding 2012). Photo by M. Holloran

The study area encompassed the overlap of two datasets (Figure 1): an updated DART dataset for the Upper Colorado River Basin (building on Nauman et al., 2017) and well pad data compiled for the Wyoming Landscape Conservation Initiative area (Garman & McBeth, 2014, 2015). This area encompassed 44,339 km^2^ of mostly intermountain basins characterized by cold, semi‐desert conditions where snow and early spring rain produce most annual precipitation (Bowen et al., 2008). Elevation ranged from 1842 m to 4194 m (U.S. Geological Survey, 2017), and ecosystems within the study area consisted of sagebrush, grassland, salt desert, and cushion plant communities. Southwesterm Wyoming also contains substantial reserves of fossil fuels (Biewick & Wilson, 2014), and development of these resources could further impact sagebrush‐dependent wildlife (Copeland et al., 2009; Garman, 2018; Heinrichs et al., 2019).

## 3 METHODS

### 3.1 Well pad data

We created timestamped well pad data by joining production start and end years reported by the Wyoming Oil and Gas Conservation Commission (https://wogcc.wyo.gov/, accessed June 9, 2017) with hand‐digitized well pads within the study area (Garman & McBeth, 2014, 2015; Monroe et al., 2020). Well pad spatial data extended up to 2012 so we excluded any wells initiated after 2012. For each pad, we determined the likely apparent reclamation year and drilling year (Monroe et al., 2020), with the difference indicating production duration. We retained well pads with ≥1 years of production and with >1 years of remote sensing data following apparent reclamation to model trends in sagebrush cover (see Statistical analyses).

### 3.2 Remote sensing data

We used a remote sensing product characterizing sagebrush cover over time and across the study area (Homer et al., 2020; Rigge et al., 2019). This product was developed from a 2016 baseline map for percent cover of sagebrush based on high‐resolution satellite imagery (2‐m Digital Globe/World View II; *n* = 331 sites trained on ocular estimation, 2013–2017), 2013–2018 Landsat 8 imagery, and an additional pool of field measurements collected at a 30‐m scale using two 30‐m transects (from 5382 locations, 2013–2017) distributed across the Western United States (Rigge et al., 2020). From this baseline map, a record of annual sagebrush cover (1985−2018, excluding 2012) was estimated at 30‐m resolution with summer and fall imagery from Landsat 5 to 8 using a change vector and change fraction approach and regression tree models (Rigge, Shi, et al., 2019). Training data for the 1985–2018 predictions were derived from areas and times where no spectral change occurred relative to the 2016 base year, while 2016 cover values were applied to unchanged areas. A series of post‐processing methods was applied to ensure accurate post‐burn trajectories and reduce noise (detailed in Rigge, Shi, et al., 2019). Sagebrush cover estimates consisted mostly of big sagebrush (*Artemisia tridentata* Nutt), including primarily Mountain (*A*.* t*. ssp. *vaseyana*) and Wyoming (*A*.* t*. ssp. *wyomingensis*) subspecies, with less abundance of low sagebrush (*A*.* arbuscula*), black sagebrush (*A*.* nova*), three tip sagebrush (*A*.* tripartita*), silver sagebrush (*A*.* cana*), and basin big sagebrush (*A*.* t*. ssp. *tridentata*). Areas with non‐rangeland cover were masked including urban, agriculture, forest, open water, and elevations >approximately 2700 m (Rigge et al., 2020). For each well pad (*i*) and year (*t*), we calculated mean sagebrush cover in the pad area (pad*_it_*) and respective reference pixels (ref*_it_*; see DART process).

### 3.3 DART process

We used an updated version of DART that relied on new soil mapping data available for the Upper Colorado River Basin (Appendix S1, Table S1; Nauman & Duniway, 2020), where an average of 0–60 cm soil electrical conductivity (EC) replaced the Landsat minerology layers in previous DART applications (Nauman & Duniway, 2016). Soil EC measures salinity and is a key determinant of composition and behavior of vegetation communities regionally (Duniway et al., 2016). Additionally, the underlying raster covariates from Nauman and Duniway (2020) were used in DART to create an expanded soil particle size class map (summarizing soil texture, depth, and rock content) consistent with Nauman and Duniway (2016). Reference pixels (30‐m resolution) were chosen from pixels outside of the well pad within a 2‐km radius to balance our objectives of identifying ecologically similar references and tracking climate histories similar to the well pad while also selecting a sufficient sample of reference pixels. We selected reference pixels using the following four steps (Appendix S1, Figure S1): (1) eliminate masked areas (see below), (2) subset to pixels with a particle size class seen in the well pad area, (3) subset to pixels within 5% of the minimum and maximum soil EC in the well pad area, and (4) of the remaining pixels, pick the 100 most similar pixels based on topographic multivariate similarity of remaining candidates to all pixels of a given well pad. Topographic similarity was calculated using Gower's distance (Gower, 1971; van der Loo, 2017) from a broad set of digital elevation model (DEM) terrain metrics as described in Nauman and Duniway (2016). The result was a selection of 100 reference pixels within 2 km that were most similar in soil and topographic properties to pixels in the well pad area.

We applied several masks to exclude areas that were unlikely to support reference conditions for sagebrush ecosystems (summarized in Appendix S1, Table S1). These masked areas included burned areas, irrigated land and reservoirs, wind turbines, coal mines, other well pads, roads, building footprints, and local disturbance data. We also excluded areas subjected to vegetation treatments reported in the Land Treatment Digital Library (Pilliod & Welty, 2013), including exclosures (*n* = 3), chemical treatments (*n* = 24), prescribed fire (*n* = 57), seedings (*n* = 33), and other vegetation/soil manipulations (*n* = 45). Additionally, we excluded pixels where 2011 National Land Cover Dataset (NLCD) classes indicated open water, development, pasture/hay, and cultivated crops (Jin et al., 2013). Finally, we examined whether a more general approach to identifying reference pixels could replace the more detailed masks available only within our study area using only masks based on national datasets extending beyond our study area. This more general mask included NLCD classes, burned areas, roads, building footprint, vegetation treatments, and other well pad locations but not datasets for irrigated land and reservoirs, wind turbines, coal mines, and local disturbance.

To increase the likelihood that well pads and reference pixels were located in sagebrush ecosystems, we excluded pads from our sample based on several criteria. First, we excluded well pads with <100% overlap with sagebrush remote sensing data. Second, we excluded well pads if mean sagebrush cover among reference pixels was ≤5.9% in any year following apparent reclamation, corresponding with the root mean square error for sagebrush estimates when compared with independent high resolution data (Rigge et al., 2019) and therefore potentially lacking sagebrush. Third, we used a LANDFIRE dataset for Existing Vegetation Type (LF 2.0.0; Rollins, 2009) with a crosswalk to Society of American Foresters‐Society for Range Management cover types to retain pads if at least one reference pixel was classified as “Mountain Big Sagebrush,” “Wyoming Big Sagebrush,” “Sagebrush‐Grass,” or “Big Sagebrush‐Bluebunch Wheatgrass.”

### 3.4 Covariates affecting recovery

We acquired several datasets characterizing biotic and abiotic factors that may account for variation in sagebrush recovery (Monroe et al., 2020). We used daily precipitation and temperature estimated at each well pad from 1 km Daymet climate data (1986−2018; Thornton et al., 1997, 2016). Minimum temperatures and precipitation during winter and spring can impact sagebrush establishment and survival (Apodaca et al., 2017; Brabec et al., 2017; Germino & Reinhardt, 2014; Maier et al., 2001; Monroe et al., 2020), so for each year we calculated precipitation totals and mean minimum temperatures during winter (December–February) and spring (March−May). Sagebrush growth also may be related to maximum temperatures and annual precipitation (Apodaca et al., 2017), so we calculated total precipitation and mean maximum temperature by water year (October–September). Other seasons and indices of moisture availability could be considered; however, here we aimed to identify several plausible factors and evaluate their relative effects on projected sagebrush recovery. To account for missing estimates of sagebrush cover in 2012, we averaged weather covariates from 2011 to 2013.

Additionally, we quantified static site conditions of soils and elevation. We determined mean elevation of each well pad using a 1/3 arc‐second DEM (U.S. Geological Survey, 2017) with hydrological corrections from Optimized Pit Removal software (Soille, 2004). Soil characteristics may influence growth and survival of sagebrush, including soil texture, soil depth, and salinity, among other factors (Barnard et al., 2019; Germino & Reinhardt, 2014; Renne et al., 2019). We therefore used a recent soil properties dataset (Nauman & Duniway, 2020) to represent several important soil variables. For each pad, we extracted mean values for percent sand (indicator of texture) and EC (indicator of salinity) from the surface down to 60 cm. We also used results from Nauman and Duniway (2020) to estimate depth to restrictive layer (primarily bedrock) as an indication of the physical soil profile for water capacity and rooting zone. We excluded seven well pads that lacked soil property estimates. Like climate factors, many physical and biochemical soil properties may affect plant growth, and here we sought to identify a set of variables to indicate soil–recovery relationships rather than evaluate all possible soil characteristics. Finally, we recorded each well pad's size (ha), years since apparent reclamation, and production duration. We acquired and formatted all data with the packages *daymetr* v. 1.4 (Hufkens, 2019), *raster* v. 3.4–5 (Hijmans, 2020), and *rgdal* v. 1.5–23 (Bivand, 2021) in R v. 3.6.3 (R Development Core Team, 2020).

### 3.5 Statistical analyses

At each well pad *i* and year *t* (1986−2018, excluding 2012), we modelled variation in the natural log of mean sagebrush cover (log[pad*_it_*]) in response to covariates using generalized additive models (GAM), which permit modelling both linear and nonlinear variation over space and time (Monroe et al., 2020; Wood, 2017). We excluded pad‐by‐year samples with 0% mean sagebrush cover for the pad area in the current or previous year to facilitate modeling cover on the log scale (Tredennick et al., 2016). We fit linear effects of well pad size (area*_i_*); soils (sand*_i_*, ec*_i_*, resdt*_i_*); temperature (temp*_it_*), precipitation (precip*_it_*), elevation (elev*_i_*), and their interactions; and smooth terms for years since apparent reclamation (time*_it_*) and production duration (duration*_i_*) based on penalized regression splines. Prior to analysis, we standardized continuous covariates by subtracting the sample mean and dividing by the sample standard deviation. In addition to direct effects on vegetation, soil texture and depth also can affect water infiltration and soil water storage capacity, and therefore we specified interactions between precip*_it_* and sand*_i_* and between precip*_it_* and resdt*_i_* to account for potential soil–climate relationships. We also specified a spatio‐temporal dependence term from the natural log of the previous year's well pad sagebrush cover (log[pad*_it_*_‐1_]), approximating a Gompertz population model (Ives et al., 2003). To account for rates of change in sagebrush cover relative to each dynamic reference, we included a covariate for the natural log of mean sagebrush cover in ref*_it_*. Because we lacked annual estimates of sagebrush cover for 2012, we used an offset for differences in time intervals between years (interval*_t_*). Finally, we specified a tensor product of thin plate regression splines (*f_te_* [*x_i_*, *y_i_*]) for the centroid location of each well pad (with geographic coordinates *x_i_* and *y_i_*; Hefley et al., 2017; Wood, 2017) to accommodate additional, unmodelled spatial variation across the study area. We determined collinearity among linear effects was acceptable based on variance inflation factors (VIF < 3.0; Zuur et al., 2010). Concurvity can indicate that smooth terms are approximated by one or more other smooth terms in a model (Wood, 2008), but observed concurvity was low to moderate among smooth terms and quantiles (0.09–0.53).

Instead of fitting models to the mean of the response variable, we estimated parameters independently at different quantiles of the response with the package *qgam* v. 1.3.2 (Fasiolo, 2020) in R. As an extension of GAM methodology developed previously (Wood, 2017), this package efficiently estimates smoothing functions at each quantile via an empirical Bayesian approach by minimizing the Extended Log‐F (ELF) loss with a belief‐updating framework (Fasiolo et al., 2020). We obtained standard errors for regression coefficients from the model variance/covariance estimated with Bayesian calibration (Fasiolo et al., 2020). We considered quantiles from τ = 0.1 to τ = 0.9 at intervals of 0.1, and we modelled sagebrush cover on well pads $Q_{\log\left({\text{pad}}_{\mathrm{it}}\right)}$ at the τth quantile given data X with the following model:

$$\begin{array}{cc} Q_{\log\left({\text{pad}}_{\mathrm{it}}\right)}\left(\tau |X\right) & =\beta_{0}\left(\tau \right)+\beta_{1}\left(\tau \right){\text{area}}_{i}+\beta_{2}\left(\tau \right){\text{sand}}_{i}+\beta_{3}\left(\tau \right){\text{ec}}_{i}+\beta_{4}\left(\tau \right){\text{resdt}}_{i}+\beta_{5}\left(\tau \right){\text{temp}}_{\mathrm{it}}\\ & +\beta_{6}\left(\tau \right){\text{precip}}_{\mathrm{it}}+\beta_{7}\left(\tau \right){\text{sand}}_{i}\times {\text{precip}}_{\mathrm{it}}+\beta_{8}\left(\tau \right){\text{resdt}}_{i}\times {\text{precip}}_{\mathrm{it}}+\beta_{9}\left(\tau \right){\text{elev}}_{i}\\ & +\beta_{10}\left(\tau \right){\text{temp}}_{\mathrm{it}}\times {\text{precip}}_{\mathrm{it}}+\beta_{11}\left(\tau \right){\text{temp}}_{\mathrm{it}}\times {\text{elev}}_{i}+\beta_{12}\left(\tau \right){\text{precip}}_{\mathrm{it}}\times {\text{elev}}_{i}\\ & +\beta_{13}\left(\tau \right){\text{temp}}_{\mathrm{it}}\times {\text{precip}}_{\mathrm{it}}\times {\text{elev}}_{i}+\beta_{14}\left(\tau \right)\log\left({\text{pad}}_{it‐1}\right)+\beta_{15}\left(\tau \right)\log\left({\text{ref}}_{\mathrm{it}}\right)\\ & +f_{\mathrm{te}}\left(\tau \right)\left(x_{i},y_{i}\right)+f_{s}\left(\tau \right)\left({\text{time}}_{\mathrm{it}}\right)+f_{s}\left(\tau \right)\left({\text{duration}}_{i}\right)+\text{offset}\left(\log\left({\text{interval}}_{t}\right)\right). \end{array}$$

We fit three models with either winter, spring, or annual weather covariates and compared their support at each quantile using Akaike's Information Criterion (AIC; Akaike, 1973). To facilitate interpretation of covariate relationships, we selected five quantiles to represent a range in recovery trends (τ = 0.1, 0.2, 0.5, 0.8, and 0.9). We evaluated covariate relationships by predicting well pad sagebrush cover at each quantile with increasing values of each covariate while maintaining other covariates at their sample mean, including pad*_it_*_‐1_ = 7.0% and ref*_it_* = 12.8%. The above analyses were based on DART reference pixels identified after applying local and general disturbance masks, but we also compared results from reference pixels after only applying general masks to evaluate the utility of our approach beyond the study area.

To evaluate predictive performance of our models, we used split conformalized quantile regression (Romano et al., 2019). For this approach, we created two within‐sample training datasets by excluding approximately 10% of the well pads in the local dataset (*n* = 120 well pads) or three randomly selected years (1987, 2000, and 2011) to evaluate spatial or temporal prediction errors, respectively. We then predicted log(pad*_it_*) for the withheld (test) data and computed conformity scores (*E*(τ)) as:

$$E(\tau )={\hat{Q}}_{\log\left({\text{pad}}_{\mathrm{it}}\right)}\left(\tau |X\right)‐\log\left({\text{pad}}_{\mathrm{it}}\right)\;\text{for}\;\tau <0.5,\;\text{and}$$

$$E(\tau )=\log\left({\text{pad}}_{\mathrm{it}}\right)‐{\hat{Q}}_{\log\left({\text{pad}}_{\mathrm{it}}\right)}\left(\tau |X\right)\;\text{for}\;\tau \geq 0.5.$$

We determined the proportion (*p*) of *E*(τ) > 0.0 for τ < 0.5, and 1 – *p* for τ ≥ 0.5, where differences in *p* relative to their respective quantile indicate predictive lack‐of‐fit. We also calculated conformalized quantile predictions by determining the τth empirical quantile, QE_pred_(τ), of *E*(τ) for τ ≥ 0.5 and 1 − τth empirical quantile for τ < 0.5. QE_pred_(τ) is then added to ${\hat{Q}}_{\log\left({\text{pad}}_{\mathrm{it}}\right)}\left(\tau |X\right)$ for τ ≥ 0.5 or subtracted for τ < 0.5. Comparing conformalized predictions to ${\hat{Q}}_{\log\left({\text{pad}}_{\mathrm{it}}\right)}\left(\tau |X\right)$ thereby indicated the marginal degree of bias in our spatial or temporal predictions.

We used models fit at each quantile to project the number of years until recovery (pad*_it_* ≥ ref*_i_*) for pixels across the study area that recovered within 100 years, and relative recovery after 100 years ($\frac{{\text{pad}}_{i100}}{{\text{ref}}_{i}}\times 100\%$) for pixels that did not recover (recovered pixels had recovery fixed at 100%). At finer scales (such as for individual disturbance areas), DART can be used to identify relevant references and inform recovery projections. In this case, however, it was not practical to use DART to identify reference pixels for each individual pixel across the study area. Instead, we created a reference for each pixel (ref*_i_*) by first identifying the temporal 95th percentile of each pixel across the 33‐year time series of sagebrush cover, which should indicate the pixel's potential for sagebrush cover while avoiding annual anomalies in estimates from remote sensing products (temporal error). We then calculated a 135‐m radius average of sagebrush cover centered at each pixel (equivalent to a 9‐pixel diameter circle) to reduce influence from spatial errors and consider local contexts. Thus, these projections were based on models fit with references identified by DART but applied to a landscape where references were identified by the approach described above. We removed pixels with ref*_i_* ≤5.9% to avoid projections in non‐sagebrush ecosystems. We assumed 30‐year averages (1989−2018) of temperature and precipitation for each pixel and iteratively predicted pad*_it_* given pad*_it_*_‐1_, ref*_i_*, elevation, and soil properties across the study area (Appendix S1, Figure S2). For the first year (*t* = 1), we assumed sagebrush cover at the pad in the previous year was 1% of the paired reference (pad*_i_*_0_ = 0.01 × ref*_i_*). We further assumed sample mean values for pad size and production duration, whereas years since apparent reclamation increased over time. As a measure of model selection uncertainty, for each quantile we calculated root mean squared error (RMSE) between projections of percent recovery and years to recovery from the best‐supported model and projections from models fit with the other two weather covariates.

For a practical application, we used our models to project recovery of sagebrush in greater sage‐grouse habitat. We first delineated landscape areas that were previously identified as nesting and summer (i.e., late brood‐rearing) habitat for sage‐grouse (Fedy et al., 2014). For pixels in each habitat type, we determined the 95th percentiles in sagebrush cover (as described above) and used the median across pixels as a recovery threshold: 16% and 18% sagebrush cover for nesting and summer habitat, respectively. Although representing a different scale (30‐m pixels vs. transect‐level measurements), these values are consistent with thresholds recommended in the Sage‐grouse Habitat Assessment Framework (15–25% cover for nesting and 10–25% cover for summer; Stiver et al., 2015). We repeated the projection exercise described above to determine the number of years to reach each sage‐grouse habitat threshold and percent recovery after 100 years (relative to each threshold, rather than the reference).

## 4 RESULTS

We analyzed records data from 1200 well pads and sagebrush cover 1986–2018 (19,558 pad by year samples). Time since apparent reclamation ranged 1–98 years (median = 13 years) and production duration ranged 1–90 years (median = 10 years). The annual weather model was almost always better supported (based on AIC) than models with other weather covariates (Table 1). Including a term for spatio‐temporal dependence of sagebrush cover on well pads in the prior year (*t*–1) substantially increased model support compared to the same model but without a spatio‐temporal dependence term. We also noted greater support for models with the paired reference covariate. Based on these results, we interpreted covariate relationships from the full annual weather model and compared projections from winter and spring weather models. Conformal score distributions and conformalized quantile predictions indicated predictive lack‐of‐fit and slight overestimates in spatial test data for τ ≥ 0.5, whereas temporal test data indicated slight underestimates in temporal test data, particularly for τ ≤ 0.5 (Appendix S1, Table S2, Figure S3).

**TABLE 1:** Generalized additive models fit to different quantiles of sagebrush (*Artemisia* spp.) cover on former oil and gas well pads in southwestern Wyoming, USA, ranked by Akaike's Information Criterion (AIC)

Quantile	0.1	0.2	0.3	0.4	0.5	0.6	0.7	0.8	0.9
Full									
Annual weather	**44486.4**	19065.0	**8273.0**	**8077.8**	**5947.3**	**2773.8**	**3094.1**	6717.0	**17164.1**
Winter weather	48812.9	18951.4	9549.5	10946.2	7557.7	3662.1	3375.3	6803.3	17844.7
Spring weather	47290.3	**18660.9**	8877.5	10038.1	6876.1	3649.1	3258.9	**6624.1**	17942.9
No Dependence									
Annual weather	74249.3	55331.1	44811.4	40401.2	37632.7	35723.2	35225.3	36037.2	39874.7
Winter weather	71972.3	55488.4	44685.8	40516.7	38086.2	35817.3	35430.2	36005.7	39304.9
Spring weather	71671.6	54580.8	44409.5	40197.4	37917.8	35554.0	35043.7	35991.0	39837.6
No Dependence, No Reference									
Annual weather	74604.0	56980.9	48678.7	44197.2	41898.9	40093.1	39895.2	40631.2	48158.8
Winter weather	74781.9	57974.9	49005.0	44241.1	42122.2	40102.8	39802.3	40777.5	48278.7
Spring weather	74694.9	57183.8	48966.7	44314.9	42034.9	39903.4	39735.0	40684.1	48543.2

Note

Models varied by weather covariates summarized annually (by water year, or October–September), by winter (December–February), or by spring (March–May) each year. Values in bold indicate the lowest AIC for each quantile. We report AIC for the full model and two reduced models, one without spatio‐temporal dependence terms for sagebrush cover on well pads in *t*–1 (No Dependence), and the other without both spatio‐temporal dependence terms for sagebrush cover on well pads and paired references (No Dependence, No Reference).

**FIGURE 2:**
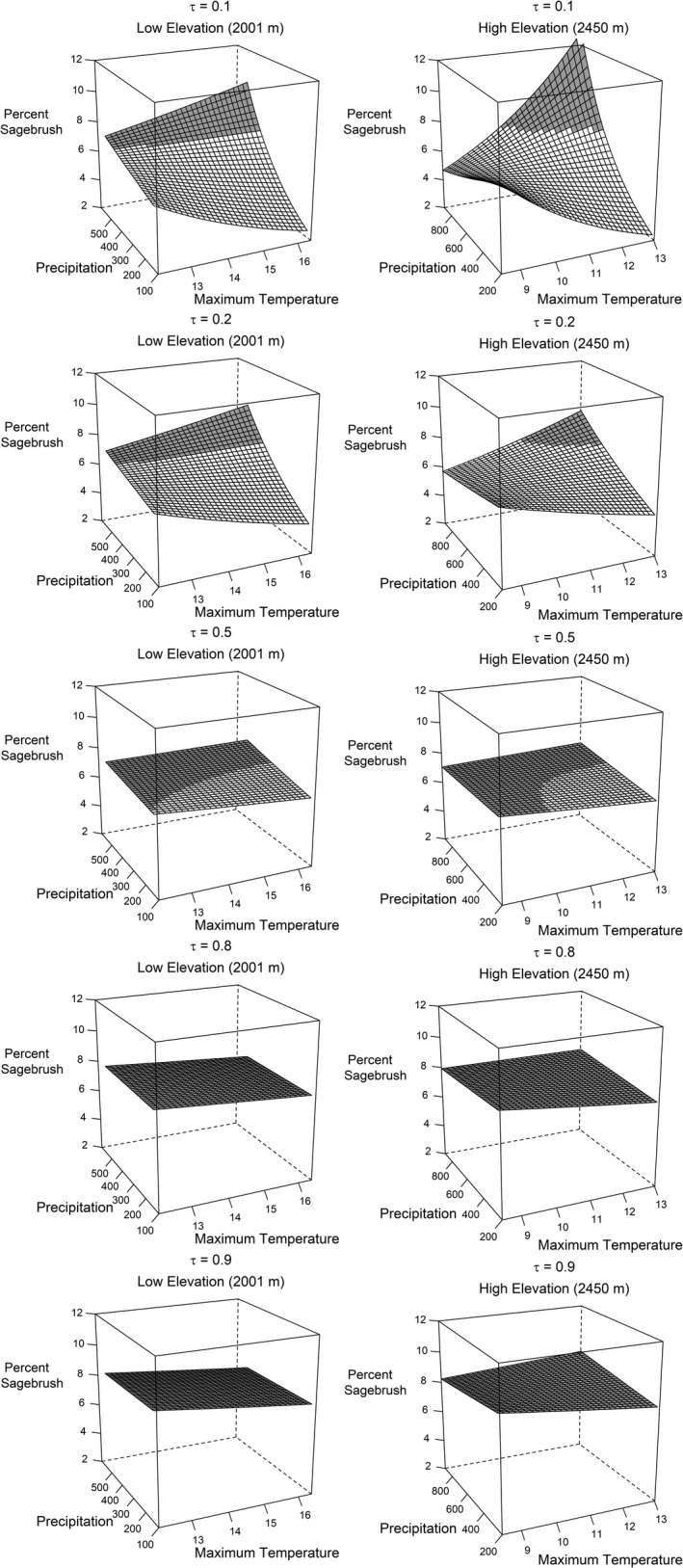
Predicted sagebrush (*Artemisia* spp.) cover with increasing annual precipitation and maximum temperature at low (left) and high (right) elevation and across quantiles (τ) for former oil and gas well pads in southwestern Wyoming, USA. Areas shaded gray denote an annual increase in sagebrush relative to sagebrush cover on well pads in the previous year (7.0%). We also assumed sagebrush cover on reference pixels in the current year (12.8%) based on a sample mean

**FIGURE 3:**
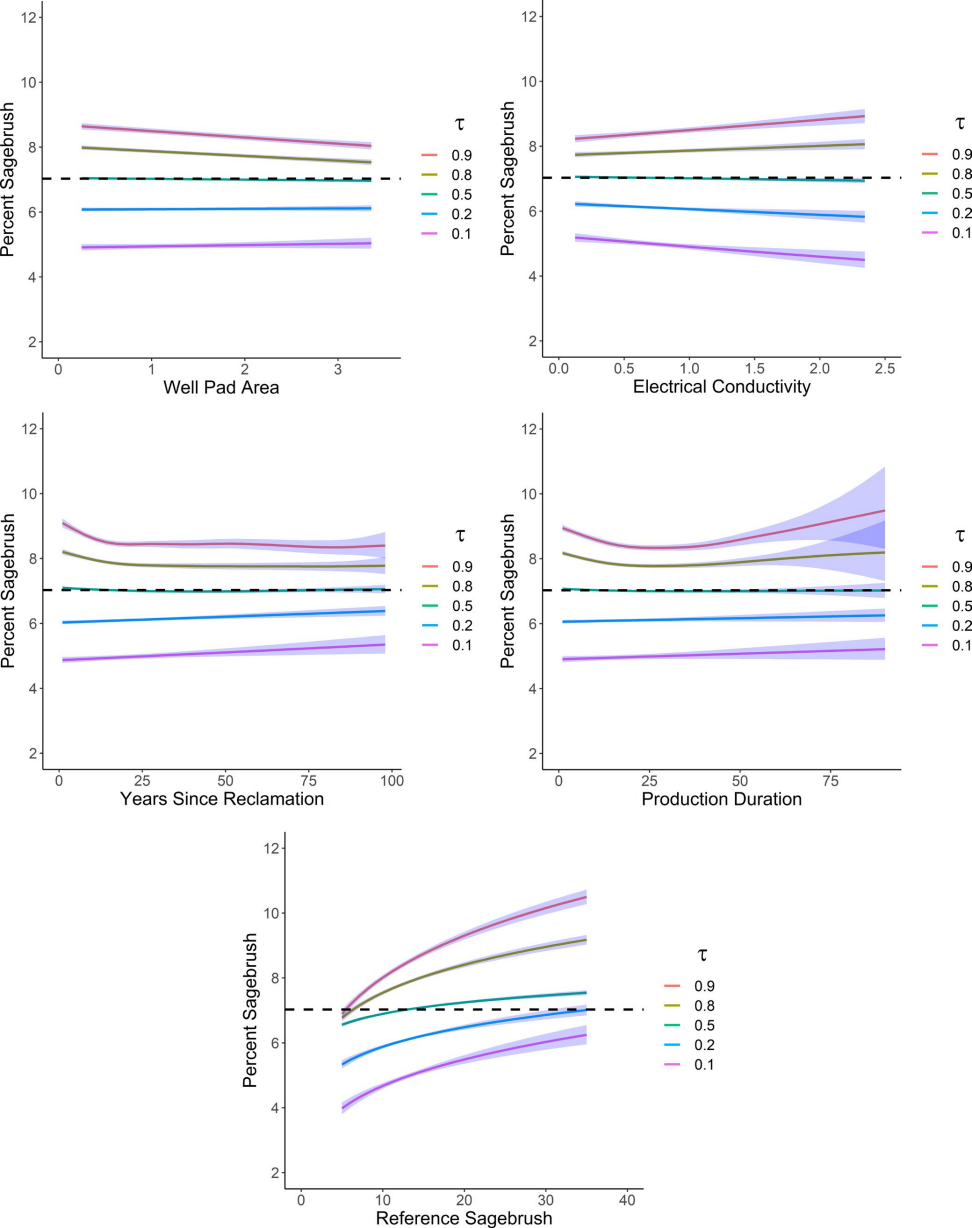
Predicted sagebrush (*Artemisia* spp.) cover ($\bar{x}$ ± 2 SE) with increasing well pad area (ha), electrical conductivity (dS m^−1^), years since apparent reclamation, production duration (years), and sagebrush cover (%) in reference pixels, and across quantiles (τ) for former oil and gas well pads in southwestern Wyoming, USA. For predictions, we assumed 7.0% sagebrush cover on well pads in the previous year (indicated by the horizontal dashed line)

**FIGURE 4:**
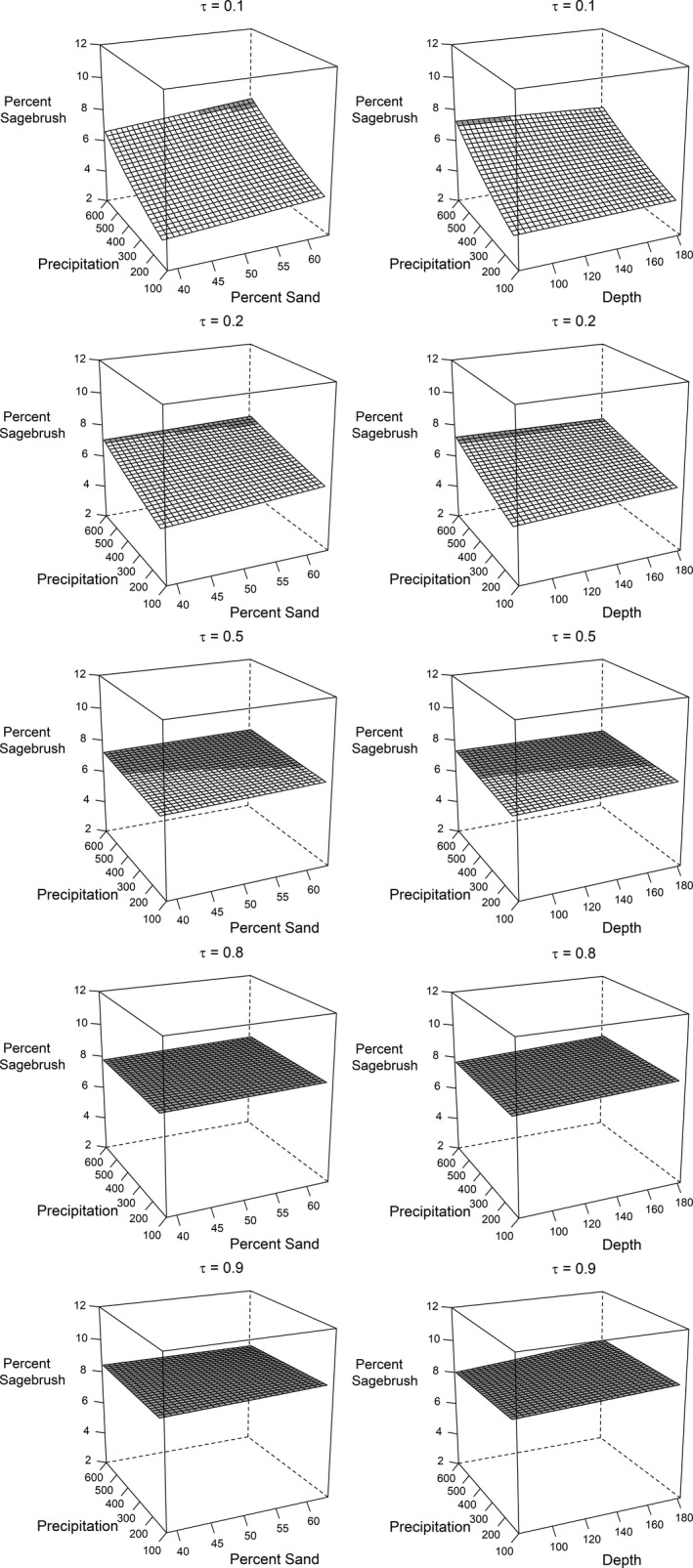
Predicted sagebrush (*Artemisia* spp.) cover from interactions between annual precipitation and percent sand (left) and depth to restrictive layer (cm; right) across quantiles (τ) for former oil and gas well pads in southwestern Wyoming, USA. Areas shaded gray denote an annual increase in sagebrush relative to sagebrush cover on well pads in the previous year (7.0%). We also assumed sagebrush cover on reference pixels in the current year (12.8%) based on a sample mean

**FIGURE 5:**
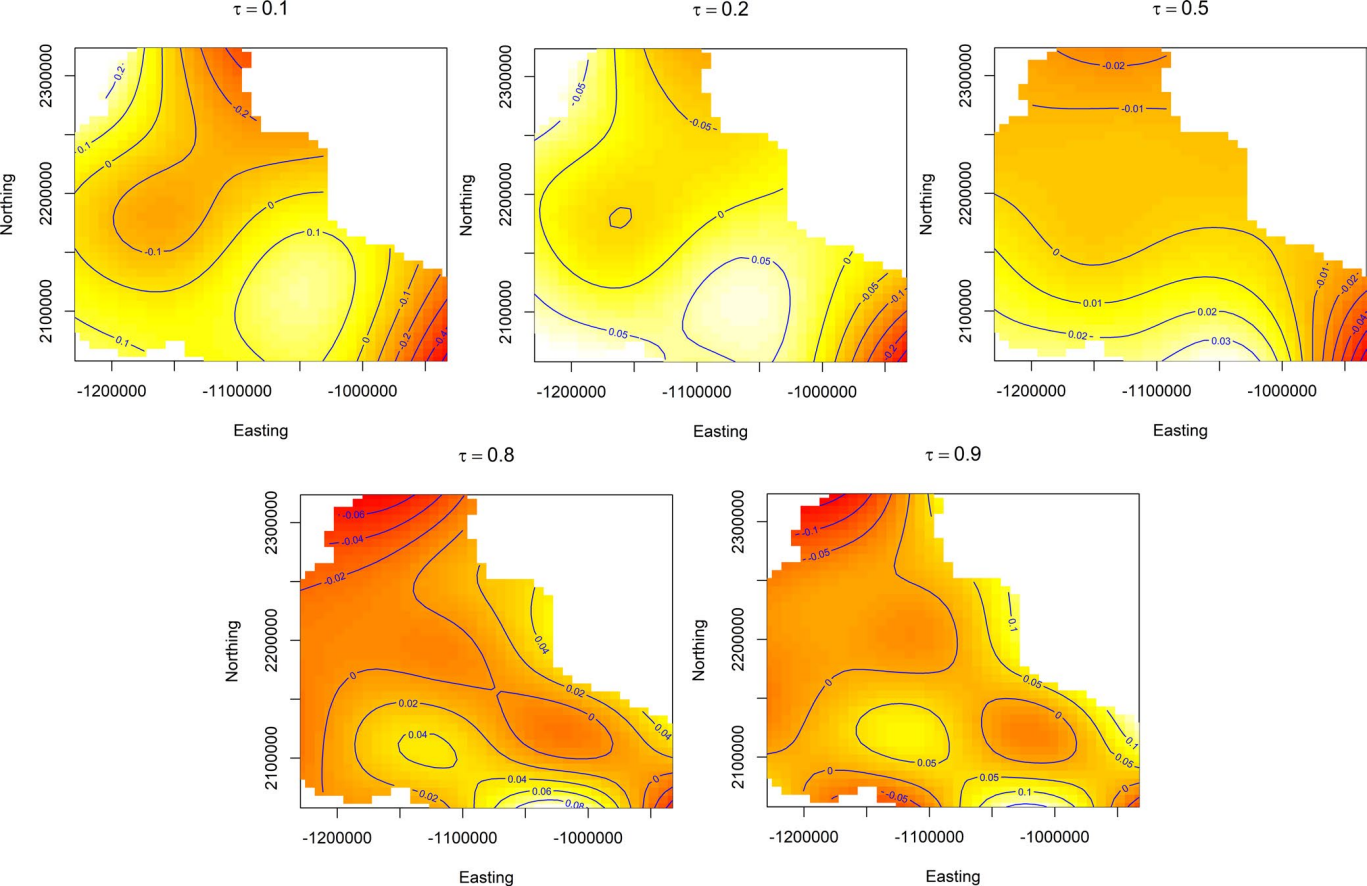
Heat maps for smoothed tensor product of location (Northing and Easting) on sagebrush (*Artemisia* spp.) cover (linear predictor scale) across quantiles (τ) for former oil and gas well pads in southwestern Wyoming, USA. Pixels values increase from red to white

Several patterns emerged across quantiles from the annual weather model, assuming all else equal. Generally, change in sagebrush cover responded more to annual variation in weather (Figure 2) than static, site‐level covariates (well pad size and soils) and smoothed temporal terms for years since apparent reclamation and production duration (Figures 3 and 4). We also estimated important spatial patterns across the study area that, depending on the quantile, tended to reduce changes in sagebrush cover in the north and southeast of the study area, and increase in south and southcentral areas (Figure 5).

The interaction between elevation, precipitation, and temperature was supported (*p* < 0.05) for most τ < 0.5 and τ > 0.7 (Table 2, Figure 2). Sagebrush cover was consistently lowest during warm and dry weather conditions across elevation and quantiles, although declines were greatest among the lower quantiles (τ < 0.5; Figure 2). Sagebrush cover increased among higher quantiles (τ > 0.5) irrespective of weather or elevation combinations. Among lower quantiles, sagebrush cover increased under warm and moist conditions at both high and low elevations, but sagebrush cover declined more under cool and moist annual conditions at high elevations than low elevations. Low quantiles also suggested greater increases in sagebrush cover than high quantiles for warm and moist conditions, particularly at high elevations, which may indicate lack‐of‐fit for such weather combinations.

**TABLE 2:** Parameter estimates (standard errors) of linear effects by quantile for the best‐supported generalized additive model of sagebrush (*Artemisia* spp.) cover (annual weather) on former well pads in southwestern Wyoming, USA

Parameter	Quantile								
	0.1	0.2	0.3	0.4	0.5	0.6	0.7	0.8	0.9
Intercept	−0.935 (0.053)	−0.462 (0.028)	−0.258 (0.015)	−0.151 (0.011)	−0.094 (0.011)	−0.040 (0.012)	0.030 (0.014)	0.113 (0.016)	0.235 (0.021)
Area	0.007 (0.006)	0.002 (0.003)	−0.001 (0.002)	−0.002 (0.001)	−0.003 (0.001)	−0.006 (0.001)	−0.010 (0.001)	−0.016 (0.002)	−0.020 (0.002)
Sand	0.041 (0.010)	0.023 (0.005)	0.014 (0.003)	0.010 (0.002)	0.009 (0.002)	0.008 (0.002)	0.007 (0.002)	0.006 (0.003)	0.006 (0.004)
Restrictive layer depth	−0.002 (0.007)	0.010 (0.004)	0.007 (0.002)	0.005 (0.001)	0.005 (0.001)	0.006 (0.002)	0.009 (0.002)	0.013 (0.002)	0.017 (0.003)
Electrical conductivity	−0.036 (0.009)	−0.016 (0.005)	−0.006 (0.003)	−0.005 (0.002)	−0.004 (0.002)	0.002 (0.002)	0.003 (0.003)	0.010 (0.003)	0.021 (0.004)
Elevation	−0.167 (0.014)	−0.059 (0.007)	−0.031 (0.004)	−0.021 (0.003)	−0.017 (0.002)	−0.016 (0.003)	−0.017 (0.003)	−0.015 (0.004)	−0.011 (0.005)
Precipitation	0.173 (0.006)	0.081 (0.003)	0.045 (0.002)	0.029 (0.001)	0.022 (0.001)	0.017 (0.002)	0.011 (0.002)	0.005 (0.002)	0.000 (0.003)
Precipitation × Sand	−0.004 (0.006)	−0.008 (0.003)	−0.005 (0.002)	−0.003 (0.001)	−0.002 (0.001)	0.000 (0.002)	0.001 (0.002)	0.000 (0.002)	−0.003 (0.003)
Precipitation × Restrictive layer depth	−0.007 (0.005)	−0.008 (0.003)	−0.003 (0.001)	−0.003 (0.001)	−0.004 (0.001)	−0.003 (0.001)	−0.002 (0.001)	0.000 (0.002)	0.003 (0.002)
Temperature	−0.181 (0.009)	−0.069 (0.005)	−0.037 (0.002)	−0.025 (0.002)	−0.021 (0.002)	−0.023 (0.002)	−0.028 (0.002)	−0.036 (0.003)	−0.042 (0.003)
Precipitation × Elevation	0.044 (0.006)	0.007 (0.004)	0.003 (0.002)	0.001 (0.002)	−0.001 (0.002)	−0.002 (0.002)	−0.001 (0.002)	0.002 (0.003)	0.006 (0.003)
Temperature × Elevation	−0.041 (0.006)	−0.009 (0.003)	−0.005 (0.002)	−0.004 (0.001)	−0.003 (0.001)	−0.003 (0.001)	−0.003 (0.002)	−0.001 (0.002)	0.003 (0.002)
Precipitation × Temperature	0.135 (0.007)	0.070 (0.004)	0.038 (0.002)	0.021 (0.002)	0.012 (0.002)	0.007 (0.002)	0.004 (0.002)	0.004 (0.002)	0.007 (0.003)
Precipitation × Temperature × Elevation	0.002 (0.003)	−0.008 (0.001)	−0.004 (0.001)	−0.002 (0.001)	−0.001 (0.001)	0.000 (0.001)	−0.001 (0.001)	0.002 (0.001)	0.003 (0.001)
log(pad*_it_*_−1_)	0.997 (0.009)	0.980 (0.004)	0.972 (0.002)	0.969 (0.002)	0.956 (0.002)	0.922 (0.003)	0.873 (0.003)	0.807 (0.003)	0.712 (0.004)
log(ref*_it_*)	0.232 (0.023)	0.140 (0.012)	0.096 (0.006)	0.072 (0.005)	0.072 (0.005)	0.089 (0.005)	0.116 (0.006)	0.156 (0.007)	0.216 (0.009)

Note

Predictions from smoothed terms are presented in Figures 3 and 5.

Sagebrush cover at well pads increased with greater reference sagebrush cover, particularly among high quantiles (Figure 3). Effects of other site‐level covariates were modest but often differed by quantile. Sagebrush cover declined slightly with pad size (mean = 1.2 ha, range = 0.2–6.3 ha) for high quantiles but not low quantiles, and sagebrush cover declined with increasing EC (mean = 0.85 dS m^–1^, range = 0.01–4.50 dS m^–1^) for low quantiles but increased slightly among high quantiles. Sagebrush cover increased slightly with years since apparent reclamation and production duration among low quantiles, but we also estimated an increase in sagebrush cover for high quantiles with recent apparent reclamation or relatively short production duration (<15 years). For several quantiles, interactions were supported between annual precipitation and depth to restrictive layer (mean = 131 cm, range = 58–200+ cm) and percent sand (mean = 52%, range = 31–75%; Table 2). Our model suggested a positive effect of precipitation among low to mid quantiles, particularly for more sandy but shallower soils (Table 2, Figure 4). This relationship was not apparent among high quantiles, where sagebrush cover increased regardless of precipitation or soils.

**FIGURE 6:**
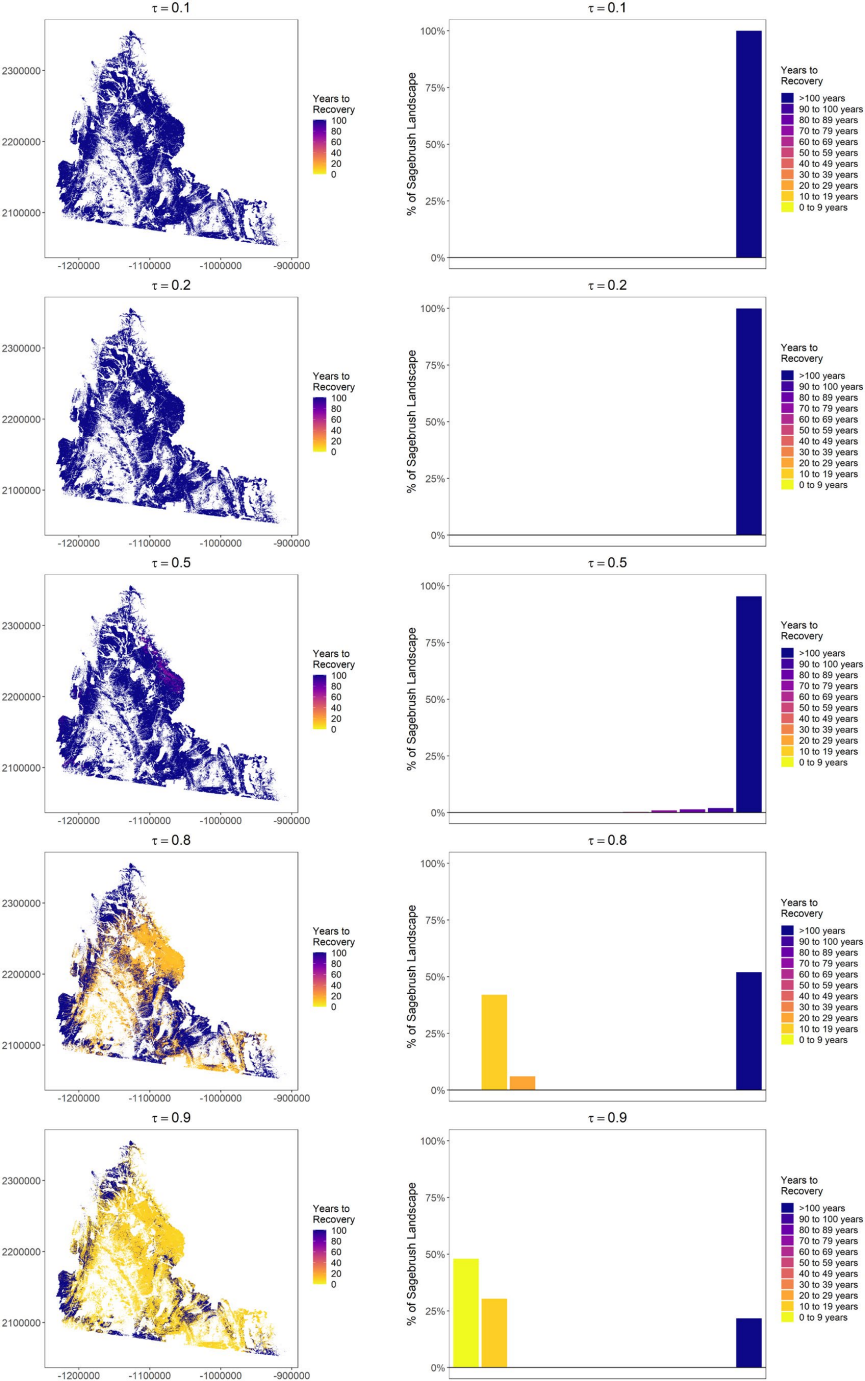
Projected time to recovery (years) of sagebrush (*Artemisia* spp.) cover across the study area (left) by quantile (τ) for former oil and gas well pads in southwestern Wyoming, USA. We also present histograms for each projection indicating the percentage of the sagebrush landscape grouped by years to recovery (right)

**FIGURE 7:**
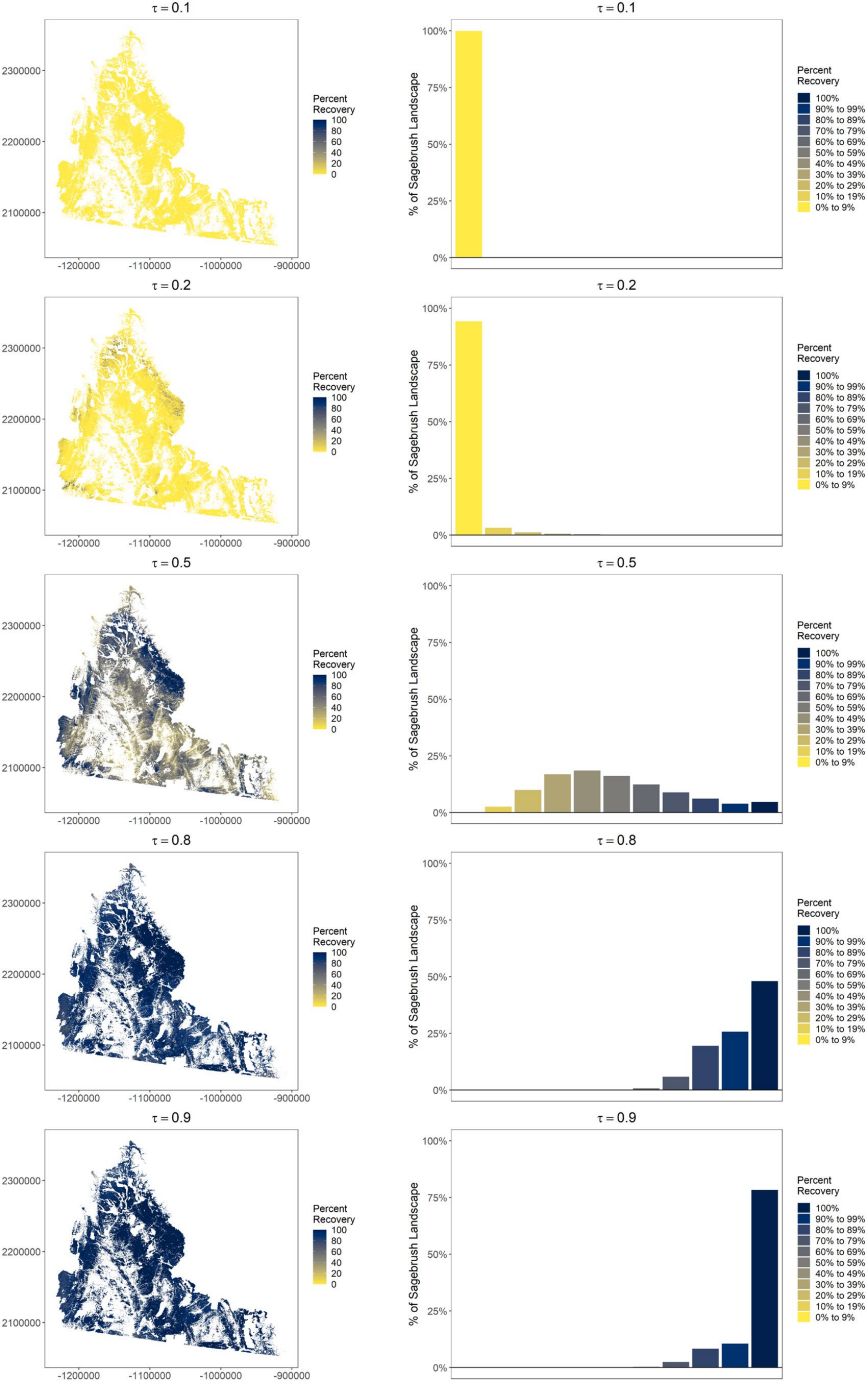
Projected percent recovery of sagebrush (*Artemisia* spp.) cover after 100 years (relative to reference areas) across the study area (left) by quantile (τ) for former oil and gas well pads in southwestern Wyoming, USA. We also present histograms for each projection indicating the percentage of the sagebrush landscape grouped by percent recovery (right)

**FIGURE 8:**
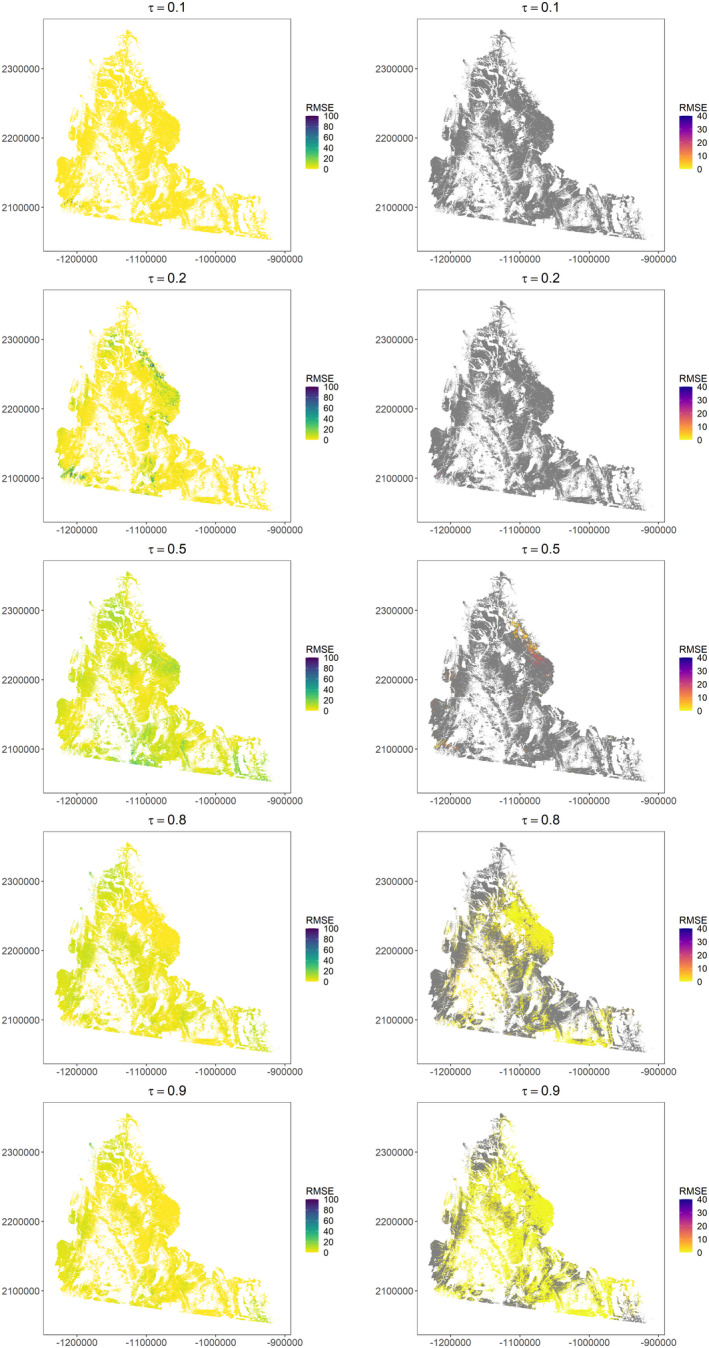
Root mean squared error for each quantile (τ) in projected percent recovery of sagebrush (*Artemisia* spp.) cover after 100 years (left) and years to recovery (right) between a model with annual weather and models with winter or spring weather. Areas in gray did not recover within 100 years based on projections from one or more models

Among low quantiles, close to none of the landscape was projected to recover within 100 years (<0.01% recovered for τ_0.1_, 0.07% recovered for τ_0.2_; Figures 6 and 7). For the median, 4.7% of the landscape recovered in 52–100 years since apparent reclamation (median τ_0.5_ = 87 years; Figure 6). High elevation areas with cool and moist conditions in the southwest and north of the study area reached >70% of their reference condition after 100 years (Figure 7). A greater proportion of the landscape recovered for high quantiles (τ_0.8_ = 48.0% recovered, τ_0.9_ = 78.3% recovered), and relatively quickly (median τ_0.8_ = 16 years, range 9−25, median τ_0.9_ = 9 years, range 5−15). Areas that did not recover still reached >32% (median τ_0.8_ = 89.9%, median τ_0.9_ = 89.8%) of their reference condition. Projections across quantiles were generally consistent (low RMSE) among much of the study area (Figure 8). However, we identified areas with higher RMSE, particularly for percent recovery projections from low to mid quantiles.

Using a more general dataset to mask potential reference pixels, we retained a larger sample (1274 well pads and 20,232 pad by year samples) than after applying the more restrictive masks with local datasets. The annual weather model was again most often best supported (Appendix S1, Table S3). Covariate relationships (Appendix S1, Figures S4–S7) were generally similar to estimates from more restrictive masks (Figures 2, 3, 4, 5). Again, we projected little recovery within 100 years for low quantiles (Appendix S1, Figures S8 and S9), with <0.01% and 0.10% of the landscape recovering in as quickly as 47 and 46 years for τ_0.1_ and τ_0.2_, respectively (τ_0.1_ median = 93 years, τ_0.2_ median = 95 years). Slightly more of the landscape recovered for the median than with the more restrictive masks (8.4% vs. 4.7%, respectively), with median recovery time of 87 years (range τ_0.5_ = 46–100 years). Most of the landscape recovered for high quantiles (τ_0.8_ = 78.4%, τ_0.9_ = 90.0%) and relatively quickly (τ_0.8_ median = 16 years, range = 9–100 years; τ_0.9_ median = 9 years, range = 5–100 years). Correlations between projections from general and more restrictive, local masks were high among mid quantiles (*r* = 0.93 and 0.99 for years to recovery and percent recovery, respectively). Projections differed somewhat for high quantiles, but generally followed the same patterns (τ_0.8_ *r* = 0.50 and 0.82 for years to recovery and percent recovery, respectively; τ_0.9_ *r* = 0.45 and 0.84 for years to recovery and percent recovery, respectively). Errors in percent recovery and recovery time projections (Appendix S1, Figure S10) also generally corresponded with broad‐scale patterns indicated by more restrictive masks (Figure 8).

Examining recovery to a threshold for achieving potential sage‐grouse nesting habitat (16% sagebrush cover), few to no pixels recovered among low quantiles (τ_0.1_ < 0.01%, τ_0.2_ = 0.37%; Appendix S1, Figure S11). We projected more pixels would recover at the median using thresholds than based on a reference (τ_0.5_ = 16.9% vs. τ_0.5_ = 10.3%, respectively), whereas fewer pixels recovered at high quantiles (τ_0.8_ = 31.3%, τ_0.9_ = 41.1% recovered relative to thresholds vs. τ_0.8_ = 51.6%, τ_0.9_ = 79.6% recovered relative to reference). We similarly observed little recovery to a threshold that should support summer habitat (18% sagebrush cover) at low quantiles (τ_0.1_ < 0.01%, τ_0.2_ = 0.40%; Appendix S1, Figure S12), higher recovery at the median (τ_0.5_ = 16.2%), and lower recovery among high quantiles (τ_0.8_ = 23.1%, τ_0.9_ = 31.9%). Patterns in percent recovery and time to recovery with thresholds were comparable to projections from across the study area based on references, albeit with less recovery (Appendix S1, Figure S13–S14).

## 5 DISCUSSION

Using ecologically relevant reference sites and a long‐term remote sensing dataset, we applied a dynamic reference approach to study and project sagebrush recovery on former oil and gas well pads. Such an application could help land managers anticipate site recovery for large geographic extents, thereby guiding restoration efforts and future disturbance. A benefit of our approach was that we could model post‐disturbance trends relative to the ecological potential of a site and annual variability of its reference. We also used quantile regression to consider heterogeneity in unmodelled factors, and quantiles could be interpreted as reflecting different levels of recovery potential at a given site and year. Some sites, for example, may experience effects of legacy land‐use, landscape context, restoration failure, ongoing disturbance, and other environmental conditions that make recovery unlikely, which were reflected in low quantiles in sagebrush cover (conditional on the predictors). Conversely, high quantiles likely represented optimistic scenarios where multiple conditions and factors converged for more favorable recovery trajectories. Indeed, we found little of the landscape was projected to recover from a potential disturbance after 100 years among quantiles less than or equal to the median response, whereas much of the landscape recovered quickly (in <25 years) among high quantiles. These results suggest recovery in some areas could be expedited if restoration conditions are favorable (e.g., Germino et al., 2018; Pyke et al., 2020; Schlaepfer et al., 2014; Shinneman & Mcilroy, 2016; Shriver et al., 2018). Additionally, we increased our spatio‐temporal sample from previous analyses (1200 vs. 375 well pads in Monroe et al., 2020) with near‐annual remote sensing products and by including well pads disturbed long before the earliest remote‐sensing imagery. Finally, we demonstrated how this approach can be applied to pressing conservation questions, such as recovery of sagebrush in sage‐grouse habitat.

Quantifying sagebrush cover on paired reference pixels over time not only offered a dynamic reference from which to evaluate recovery trajectories but also explained additional variation in sagebrush cover on former well pads, such as greater recovery among pads with high sagebrush cover at paired references. For the median, and all else being equal, reference sagebrush cover may help predict whether well pad sagebrush will decline or increase in subsequent years. Areas with high sagebrush cover in reference pixels likely corresponded with more suitable conditions for growth and resiliency, such as relatively cooler and moister soil conditions within a semi‐arid landscape (Schlaepfer et al., 2012a) which are expected to exhibit greater potential for restoration success (Chambers et al., 2014). This relationship also may reflect ability of the DART approach to identify ecologically relevant references based on a suite of soil and topographic covariates (i.e., matching of pad ecological potential; Herrick et al., 2019). However, declines in sagebrush cover among low quantiles even when reference sagebrush cover was high suggest relying on reference sagebrush cover alone is insufficient for anticipating a site's recovery rate.

Although negative effects of warm and dry conditions were apparent across quantiles, lower quantiles tended to respond more strongly to weather than higher quantiles of sagebrush cover. This result suggests among low quantiles, when site conditions, reclamation history, or both are less suitable for recovery, the outsized influence of weather warrants consideration when planning restoration. For example, warmer temperatures during periods of low moisture can stress sagebrush, reducing sagebrush establishment, growth, and survival (Schlaepfer et al., 2012b, 2014). We also estimated a positive response to warm and moist conditions among low to mid quantiles, consistent with a previous study in the region (Monroe et al., 2020) and for sagebrush in other cool climates (Kleinhesselink & Adler, 2018; Perfors et al., 2003; Renwick et al., 2017; Rigge, Shi, et al., 2019). Despite stronger responses to annual weather, however, study area projections from low quantiles seldom recovered within 100 years, which may partly reflect slight underestimates in temporal predictions we observed with conformalized predictions. These results also may indicate the importance of other, unmodelled factors determining rates of recovery. Indeed, temperature and moisture conditions near the time of planting, even at a very fine temporal resolution (e.g., daily; O’Connor et al., 2020), may be significant for subsequent recovery (Maier et al., 2001; Shinneman & McIlroy, 2016; Shriver et al., 2018; Ziegenhagen & Miller, 2009). We also lacked data on the type, timing, and occurrence of reclamation practices applied to former well pads (Monroe et al., 2020). For example, seed depth is critical to persistence and germination of sagebrush (Jensen et al., 2001; Schlaepfer et al., 2014), and success from reseeding is often low (Davies et al., 2013; Shaw et al., 2005). Lack of recovery also may indicate transitions to alternative or degraded states. Planting crested wheatgrass at disturbed sites, including well pads, for example, was once common for stabilizing soils, but this may inhibit recolonization by sagebrush (Davies et al., 2013). We caution, however, that these projections should be interpreted in the context of their underlying assumptions. We assumed 30‐year averages for weather when making projections, corresponding with climatic conditions from which we fit sagebrush trend models, but these may differ from future growing conditions (Homer et al., 2015; Palmquist et al., 2016; Renwick et al., 2017; Tredennick et al., 2016). Overall trajectories of ecological systems are unlikely to be static with a changing climate (e.g., Bradford et al., 2019), and our research highlights a need to better understand how broad‐scale variation due to a changing climate may alter plausible restoration goals (e.g., Lavorel et al., 2015; Wintle et al., 2011).

Responses from median sagebrush cover indicated a small percentage of the landscape would recover within 100 years, while much of the rest reached some level of partial recovery, often among higher elevations where conditions tend to be cooler and moister (Appendix S1, Figure S2). Mountain big sagebrush located at higher elevations may recover in 19–100 years (Baker, 2006; Lesica et al., 2007; Moffet et al., 2015; Nelson et al., 2014), whereas for Wyoming big sagebrush, occurring at lower elevations with warmer and drier conditions, recovery is estimated to take longer (Baker, 2006; Bates et al., 2020). We note, however, direct comparisons are limited because many previous estimates of sagebrush recovery are based on disturbance from fire, and recovery conditions likely differ for former well pads. For example, under current well pad reclamation practices, topsoil is removed, stockpiled, and replaced, and seed bed preparation can include recontouring, soil amendments to address salinity or low organic matter, and other intensive practices not used following wildfire (U.S. Department of the Interior‐Bureau of Land Management, 2012; U.S. Department of the Interior‐Bureau of Land Management & U.S. Department of Agriculture, 2007). Previous studies in Wyoming estimated Wyoming big sagebrush recovery in 87 or more years on individual well pads (Avirmed et al., 2015). At broader scales, 21% of the landscape was projected to recover within 60 years, whereas remaining sites may require >100 years (Monroe et al., 2020). Our current study suggests that these previous analyses (Avirmed et al., 2015; Monroe et al., 2020) may not have fully considered spatio‐temporal heterogeneity among well pads within this landscape.

Interestingly, we projected relatively fast recovery among high quantiles at lower elevation sites (warm and dry ecotype) even after accounting for lower sagebrush cover among references (lower restoration target, but also slower growth than for sites with high sagebrush cover references). While there are reports of Wyoming big sagebrush communities recovering relatively quickly (9–35 years; Wambolt et al., 2001; Shinneman & McIlroy, 2016), our projections may reflect possible factors that facilitate recovery, such as favorable establishment conditions and successful restoration practices (Pyke et al., 2020). Indeed, we estimated faster recovery with recent apparent reclamation among high quantiles, but this effect dissipated with longer time periods, possibly because fitting spatio‐temporal dependence accounted for variation in the years since apparent reclamation smooth term. Our projections also indicated areas unlikely to recover even among high quantiles, and again multiple factors not explicitly incorporated in the model (e.g., invasive annual grasses) may nevertheless drive spatial variation in recovery across the landscape. From a practical standpoint, areas not recovering among high quantiles could temper expectations for potential restoration efforts or guide disturbance away from these areas when feasible. Additionally, we identified areas where projections were sensitive to choice of weather covariate in our models, as indicated by larger RMSE, particularly among northern and southcentral areas where we lacked well pad data (Figure 1). Numerous weather and soil moisture covariates have been proposed for explaining variation in sagebrush recovery, quantified at varying spatial and temporal resolutions (e.g., from 30‐year averages [Davidson et al., 2019] to daily weather [O’Connor et al., 2020]), and further exploring these covariates with additional data may reduce projection error.

Several site‐level covariates minimally influenced sagebrush trends in this system, although the strength and direction of these effects often varied by quantile. Among low to mid quantiles, sagebrush cover increased with greater precipitation, and slightly more so with sandier and shallower soils. This result could reflect sagebrush response to permeable soils and their ability to retain accessible moisture for sagebrush growth and survival (but see Germino & Reinhardt, 2014; Renne et al., 2019; Sturges, 1989). Quantiles also responded differently to the size of former well pads and EC of soils. Among high quantiles, recovery declined with increasing well pad size, consistent with Monroe et al. (2020) and possibly reflecting short dispersal distances of sagebrush seeds (Schlaepfer et al., 2014) into former well pads from intact sagebrush. Declines among low quantiles with increasing EC may reflect salinity‐related limitations to sagebrush recovery (Cook, 1961), but this would contradict the slight increases observed with EC among high quantiles. The latter result may be a correlation with more sparse sagebrush in reference communities and thus a lower threshold for recovery (Thatcher, 1959). Differences in covariate relationships among quantiles illustrate a benefit of using quantile regression as these patterns could be useful when considering local management opportunities but may be overlooked when fitting models to the mean response.

Identifying appropriate reference sites requires matching ecological site potential, but additional information of on‐site management and disturbance history may further refine these comparisons to areas representative of well‐managed and less disturbed reference ecosystems, thereby providing a more appropriate management target (Herrick et al., 2019). For this reason, we used several relevant spatial products to mask reference pixels based on local and national datasets. Comparing inferences from a more general mask that lacked data on local conditions, we found slight differences in covariate relationships and sample size, but overall inferences were largely consistent between projections. These results suggest our approach could be applied to landscapes that lack data on local conditions, but outcomes may vary with spatial extent and sample size.

Finally, we applied our approach to project potential recovery of sagebrush in greater sage‐grouse nesting and summer habitat after similar oil and gas disturbances. We emphasize that sagebrush is only one component of sage‐grouse habitat (Fedy et al., 2014), albeit an important one for this sagebrush‐obligate species (Connelly et al., 2000). We also acknowledge that using thresholds may be counterproductive, particularly when extrapolated beyond study areas where they were first determined (Smith et al., 2020). Nevertheless, land managers often use minimum thresholds to establish objectives (Connelly et al., 2000; Stiver et al., 2015), and we determined a threshold based on a distribution of sagebrush cover estimated from habitat within the study area. Using a fixed threshold for sagebrush recovery greater than a site's reference sagebrush cover led to identifying more areas that failed to recover after 100 years for high quantiles, whereas more of the landscape was projected to recover at the median when the threshold was below a site's reference. Overall, this exercise illustrates how conservation targets for sage‐grouse could directly inform where limited resources are applied to help meet management goals; restoration success to create usable habitats. However, evaluating recovery relative to references also may indicate where restoration thresholds are less appropriate for an ecosystem.

## 6 CONCLUSIONS

Using reference sites in recovery analyses is invaluable for guiding restoration and evaluating outcomes (Bestelmeyer et al., 2009; Fick et al., 2021; Herrick et al., 2019; Nauman et al., 2015). In this study, we used the DART process to identify reference areas, and we applied these to time‐varying remote sensing maps of sagebrush cover, allowing for a dynamic characterization of reference conditions through time and ensuring continued relevance of reference sites for this slow‐growing shrub. Time‐varying sagebrush cover from paired reference pixels explained additional variation in sagebrush trends, and modelling trends for low, mid, and high quantiles offered insights into trends (and controls of trends) likely under poor, typical (median), and optimal restoration conditions. Projections indicated little of the landscape might recover after 100 years for sites with sagebrush quantiles equal to or less than the median, whereas much of the landscape recovered for high quantiles of sagebrush cover. This approach could be useful for informing management and restoration efforts in this system, such as by identifying areas unlikely to recover even under optimal conditions. Projections from this work also could be incorporated into restoration prioritization tools currently in development (Duchardt et al., 2021).

## CONFLICT OF INTEREST

The authors have no competing interests to declare.

## AUTHOR CONTRIBUTION

**Adrian P. Monroe:** Conceptualization (equal); Data curation (equal); Formal analysis (lead); Funding acquisition (lead); Investigation (equal); Methodology (equal); Project administration (lead); Resources (lead); Software (lead); Supervision (equal); Validation (equal); Visualization (lead); Writing – original draft (lead); Writing – review & editing (equal). **Travis W. Nauman:** Conceptualization (equal); Data curation (supporting); Formal analysis (supporting); Funding acquisition (equal); Investigation (supporting); Methodology (equal); Project administration (supporting); Resources (supporting); Supervision (equal); Validation (equal); Visualization (supporting); Writing – original draft (supporting); Writing – review & editing (equal). **Cameron L. Aldridge:** Conceptualization (equal); Data curation (supporting); Formal analysis (supporting); Funding acquisition (supporting); Investigation (supporting); Methodology (equal); Project administration (supporting); Resources (equal); Supervision (supporting); Validation (equal); Visualization (supporting); Writing – original draft (supporting); Writing – review & editing (equal). **Michael S. O'Donnell:** Conceptualization (equal); Data curation (supporting); Formal analysis (supporting); Investigation (supporting); Methodology (equal); Software (supporting); Supervision (equal); Validation (equal); Visualization (supporting); Writing – original draft (supporting); Writing – review & editing (equal). **Michael C. Duniway:** Conceptualization (equal); Funding acquisition (supporting); Investigation (supporting); Methodology (equal); Project administration (supporting); Resources (supporting); Supervision (equal); Writing – original draft (supporting); Writing – review & editing (equal). **Brian S. Cade:** Conceptualization (equal); Formal analysis (supporting); Investigation (supporting); Methodology (equal); Software (supporting); Supervision (equal); Validation (equal); Visualization (supporting); Writing – original draft (supporting); Writing – review & editing (equal). **Daniel J. Manier:** Conceptualization (equal); Funding acquisition (supporting); Investigation (supporting); Methodology (equal); Supervision (equal); Validation (equal); Writing – original draft (supporting); Writing – review & editing (equal). **Patrick J. Anderson:** Conceptualization (equal); Funding acquisition (supporting); Investigation (supporting); Methodology (equal); Project administration (supporting); Supervision (equal); Writing – original draft (supporting); Writing – review & editing (equal).

## Supporting information

Appendix S1

## Data Availability

Data associated with analyses in this study and all maps created are available as a U.S. Geological Survey data release (Monroe et al., 2022: https://doi.org/10.5066/P9OP5D76).
