# Supplementary material for: Assessing vegetation recovery from energy development using a dynamic reference approach
Source: Ecol Evol. 2022 Feb 17;12(2):e8508. doi: 10.1002/ece3.8508 (PMC8855019; doi:10.1002/ece3.8508)
Supplement: Supplementary file 1 — Appendix S1 [file ECE3-12-e8508-s001.docx]

Appendix S1. Supplementary tables and figures.

Table S1. Summary of datasets used in the Disturbance Automated Reference Toolset process and masking potential reference pixels. Masks were used to exclude pixels that were unlikely to support reference conditions for sagebrush in the study area, southwestern Wyoming, USA.

| Dataset | Citation | Format | Resolution | Purpose |
| --- | --- | --- | --- | --- |
| Expanded particle size class map | Nauman & Duniway (2020) | Raster | 30 m | DART |
| Topographic similarity | Nauman & Duniway (2016) | Raster | 30 m | DART |
| Burned Area Essential Climate Variable | Hawbaker et al. (2017) | Raster | 30 m | Mask |
| Irrigated land and reservoirs | Wyoming Water Development Office (2018) | Vector | - | Mask |
| Wind turbines | O’Donnell and Fancher (2010, 2014) | Vector | - | Mask |
| Coal mines | Wyoming Department of Environmental Quality (2018) | Vector | - | Mask |
| Oil and gas well pads | Garman & McBeth (2014, 2015) | Vector | - | Mask |
| Roads | Unpublished dataset (T. Fancher, USGS, pers. comm., 14 December 2020), updated from O’Donnell et al. (2014) | Vector | - | Mask |
| Building footprint | Microsoft® Maps (2019) | Vector | - | Mask |
| Wyoming Density and Disturbance Calculation Tool | Wyoming Geographic Information Science Center (2019) | Vector | - | Mask |
| Land Treatment Digital Library | Pilliod et al. (2019) | Vector | - | Mask |
| 2011 National Land Cover Dataset | Jin et al. (2013) | Raster | 30 m | Mask |

References

Garman S.L., & McBeth J.L. (2014). Digital representation of oil and natural gas well pad scars in southwest Wyoming. *U.S. Geological Survey Data Series 800*, 7 p. http://dx.doi.org/10.3133/ds800

Garman S.L., & McBeth J.L. (2015). Digital representation of oil and natural gas well pad scars in southwest Wyoming–2012 update. *U.S. Geological Survey Data Series 934*, http://dx.doi.org/10.3133/ds934

Hawbaker T.J., Vanderhoof M.K., Beal Y.-J., Takacs J.D., Schmidt G.L., Falgout J.T., Williams B., Fairaux N.M., Caldwell M.K., Picotte J.J., Howard S.M., Stitt S., & Dwyer J.L. (2017). Mapping burned areas using dense time-series of Landsat data. *Remote Sensing of Environment*, 198, 504-522. https://doi.org/10.1016/j.rse.2017.06.027

Jin, S., Yang L., Danielson P., Homer C., Fry J., & Xian G. (2013). A comprehensive change detection method for updating the National Land Cover Database to circa 2011. *Remote Sensing of the Environment*, 132, 159-175. https://doi.org/10.1016/j.rse.2013.01.012

Microsoft® Maps (2019). U.S. Buildings Footprint, v2.0. Retrieved from https://github.com/Microsoft/USBuildingFootprints (accessed December 14 2020)

Nauman T.W., & Duniway M.C. (2016). The Automated Reference Toolset: A soil-geomorphic ecological potential matching algorithm. *Soil Science Society of America Journal*, 80, 1317-1328. https://doi.org/10.2136/sssaj2016.05.0151

Nauman T.W., Duniway M.C., Villarreal M.L., & Poitras T.B. (2017). Disturbance automated reference toolset (DART): Assessing patterns in ecological recovery from energy development on the Colorado Plateau. *Science of the Total Environment*, 584-585, 476-488. https://doi.org/10.1016/j.scitotenv.2017.01.034

O’Donnell M.S., & Fancher T.S. (2010). Spatial mapping and attribution of Wyoming wind turbines. *U.S. Geological Survey Data Series 524*, https://pubs.usgs.gov/ds/524

O’Donnell M.S., & Fancher T.S. (2014). Spatial mapping and attribution of Wyoming wind turbines-2012. *U.S. Geological Survey Data Series 828*, https://dx.doi.org/10.3133/ds828

O'Donnell M.S., Fancher T.S., Freeman A.T., Ziegler A.E., Bowen Z.H., & Aldridge C.L. (2014). Large scale Wyoming transportation data: A resource planning tool. *U.S. Geological Survey Data Series 821*, 21 p. http://dx.doi.org/10.3133/ds821

Pilliod D.S., Welty J.L., & Jeffries, M.I. (2013). USGS Land Treatment Digital Library Data Release: A centralized archive for land treatment tabular and spatial data (ver. 1.0, April 2019). *U.S. Geological Survey data release*, https://doi.org/10.5066/P98OBOLS

Wyoming Department of Environmental Quality (2018). Wyoming Active Coal Mine Permit Boundaries. Retrieved from http://deq.wyoming.gov/lqd/coal/resources/chia/ (accessed May 3 2018)

Wyoming Geographic Information Science Center (2019). Wyoming Density and Disturbance Calculation Tool. Retrieved from https://ddct.wygisc.org/home.aspx (accessed May 10 2019)

Wyoming Water Development Office (2018). Irrigated land and reservoirs in Wyoming. Retrieved from https://waterplan.state.wy.us/plan/statewide/gis/gis.html (accessed October 15 2018)

Table S2. Conformal score distributions (*p*) for spatial and temporal test data and five quantiles (τ). We created a spatial test dataset by randomly excluding 10% (*n* = 120) oil and gas well pads and a temporal test dataset by randomly excluding three years (1987, 2000, and 2011), then computed conformity scores by fitting models to each quantile of the training datasets and calculating the difference between predicted and observed sagebrush (*Artemisia* spp.) cover for tests datasets. We also report quantiles of the conformal scores QE_pred_(τ), on the log scale, for spatial and temporal test datasets.

| τ | *p*_spatial_ | *p*_temporal_ | QE_pred_(τ) spatial | QE_pred_(τ) temporal |
| --- | --- | --- | --- | --- |
| 0.1 | 0.112 | 0.061 | 0.025 | –0.060 |
| 0.2 | 0.206 | 0.143 | 0.003 | –0.039 |
| 0.5 | 0.516 | 0.414 | –0.003 | 0.024 |
| 0.8 | 0.855 | 0.762 | –0.026 | 0.020 |
| 0.9 | 0.941 | 0.886 | –0.045 | 0.010 |

Table S3. Models of sagebrush (*Artemisia* spp.) cover on former oil and gas well pads in southwestern Wyoming, USA, ranked by Akaike’s Information Criterion (AIC). Models varied by weather covariates summarized annually (by water year, or October–September), by winter (December–February), or by spring (March–May) each year. Values in bold indicate the lowest AIC for each quantile. We fit models after using a general dataset to mask potential reference pixels (without local datasets on irrigated land and reservoirs, wind turbines, coal mines, and the Density Disturbance Calculation Tool).

| Model | Quantile | | | | | | | | |
| --- | --- | --- | --- | --- | --- | --- | --- | --- | --- |
|  | 0.1 | 0.2 | 0.3 | 0.4 | 0.5 | 0.6 | 0.7 | 0.8 | 0.9 |
| Annual weather (tmax) | **45642.6** | 18967.7 | **8063.3** | **7957.5** | **5583.7** | **2255.7** | **2525.1** | 6256.4 | **17430.1** |
| Winter weather (tmin) | 49017.1 | 18636.6 | 9295.5 | 10549.0 | 7278.3 | 3179.5 | 2813.2 | 6277.9 | 18292.5 |
| Spring weather (tmin) | 47764.7 | **18542.0** | 8688.3 | 9992.9 | 6662.5 | 3136.5 | 2696.0 | **6069.2** | 18340.8 |


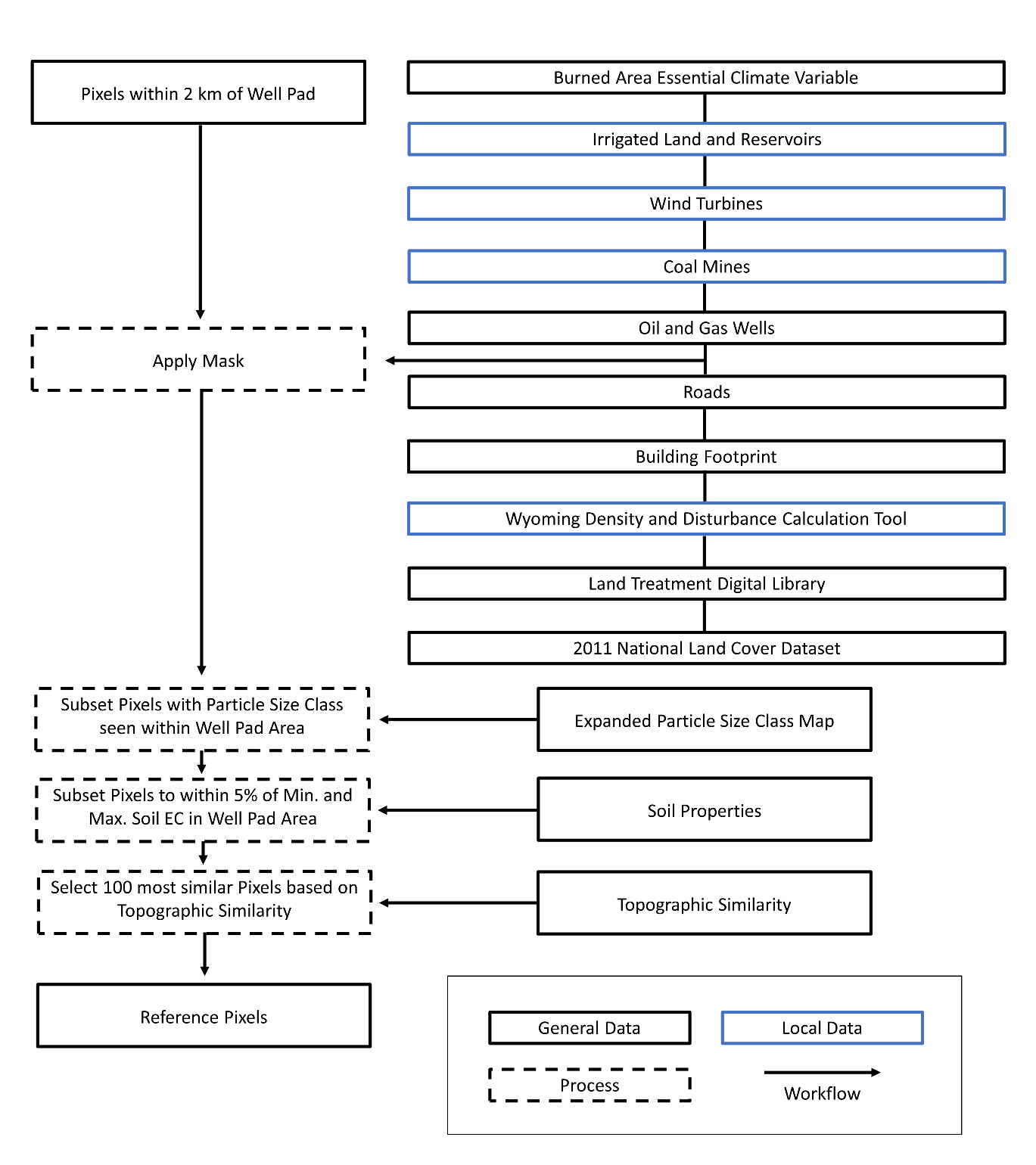


Figure S1. Overview of the Disturbance Automated Reference Toolset process for identifying reference pixels for each well pad, including necessary datasets (general and local), processing steps, and workflow. EC = electrical conductivity.


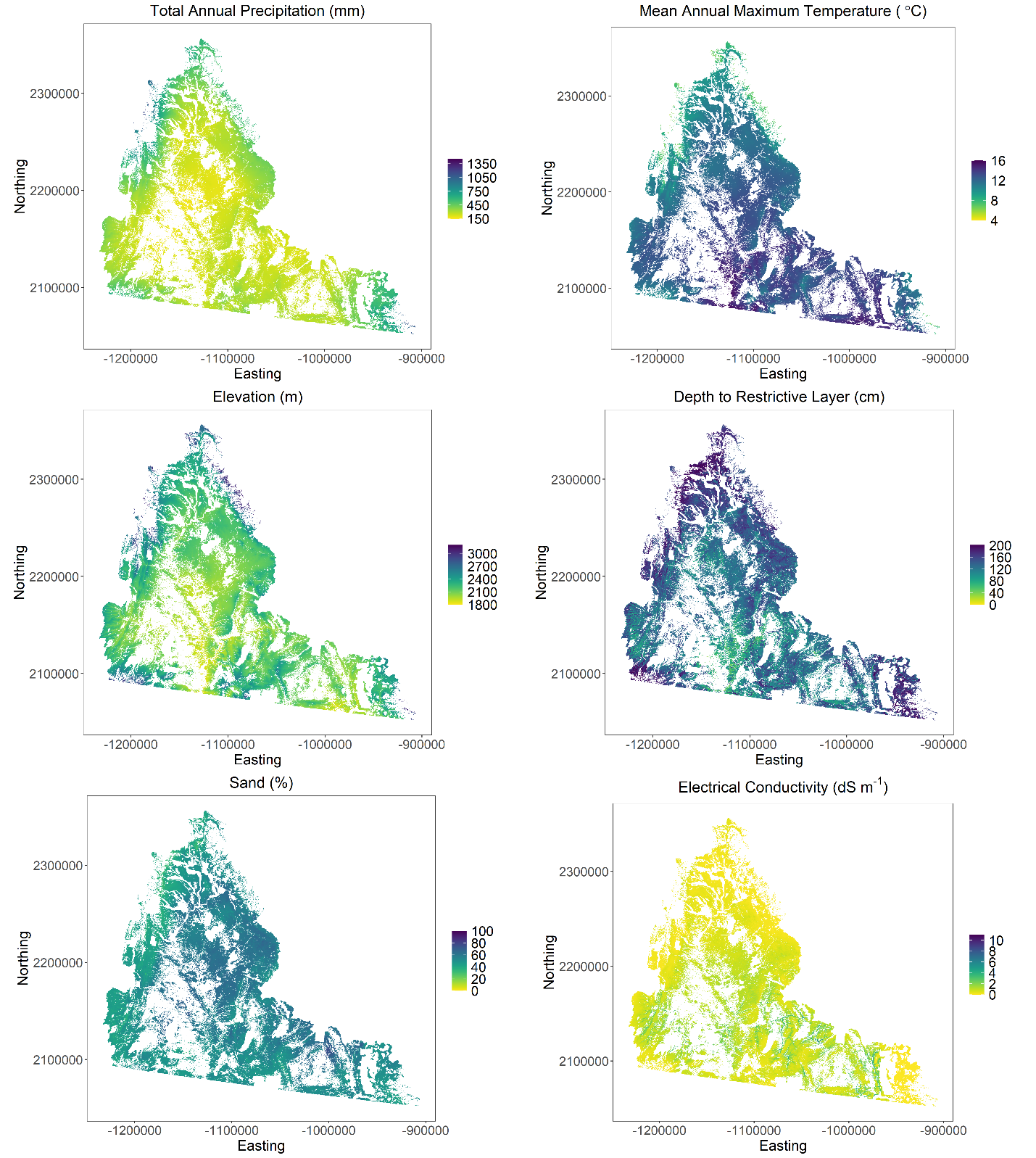


Figure S2. We used the following spatial datasets when projecting recovery of sagebrush cover at former oil and gas well pads across the study area in southwestern Wyoming, USA, including 30-year averages (1989−2018) of total annual precipitation and mean annual maximum temperature, elevation, depth to restrictive layer, and sand and electrical conductivity of soils. Pixels without color were masked or lacked sagebrush (*Artemisia* spp.) predictions.


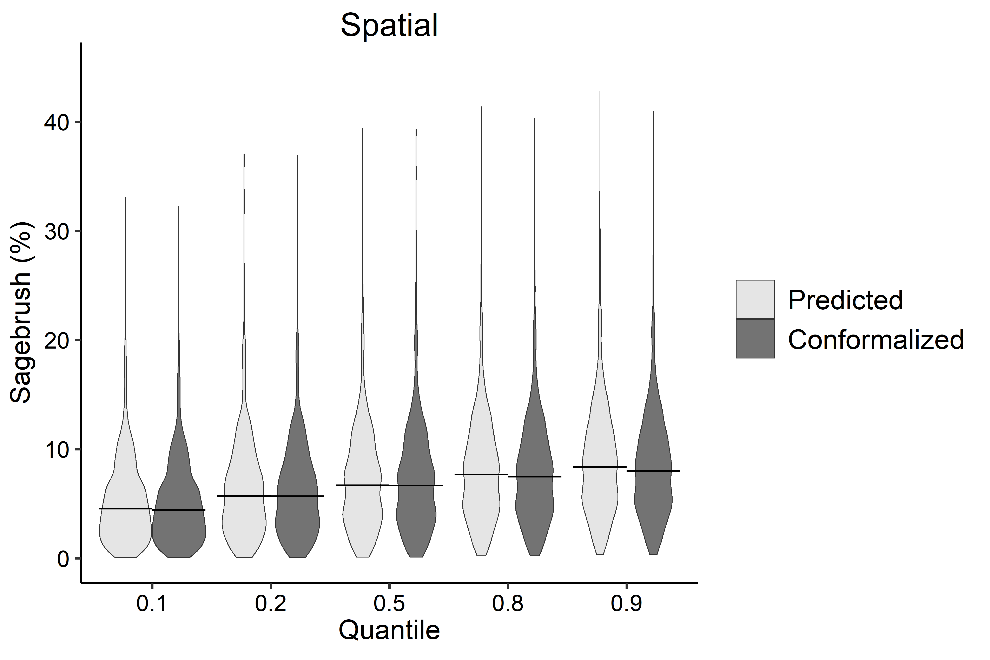

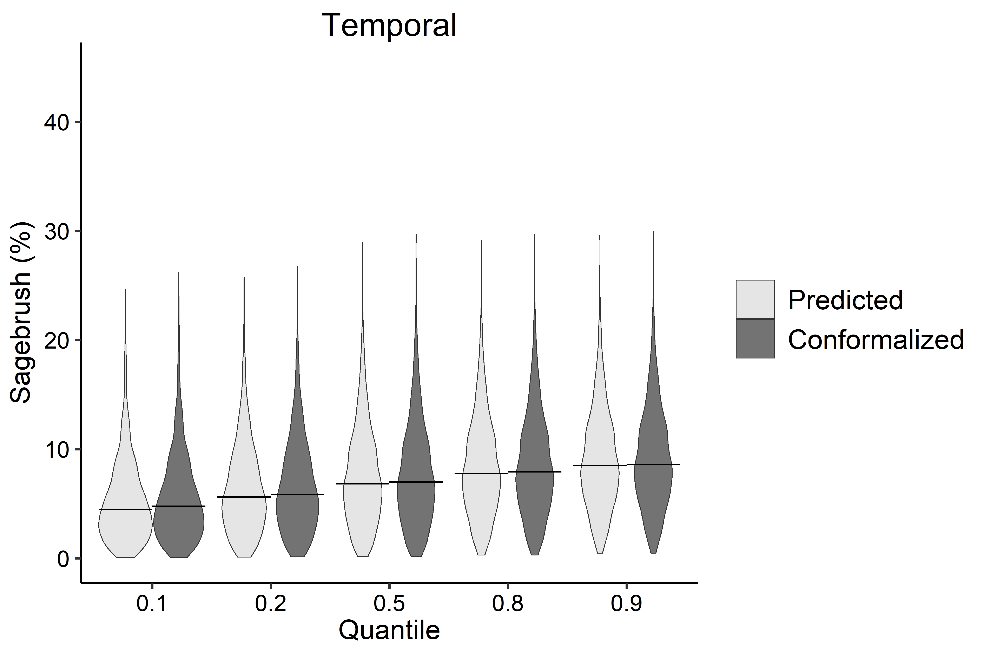


Figure S3. Violin plots (with medians indicated by horizontal bars) for predicted sagebrush (*Artemisia* spp.) cover at well pads for spatial (*n* = 2,056 pad by year samples) and temporal (*n* = 1,644) test datasets, presented alongside conformalized quantile predictions with a marginal correction based on the empirical quantile of conformity scores for each quantile.


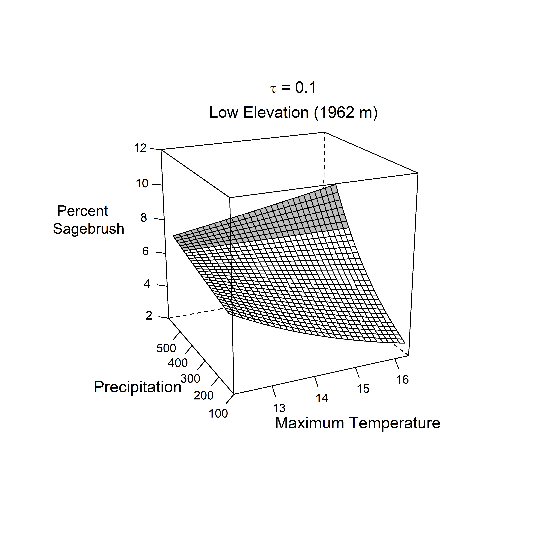

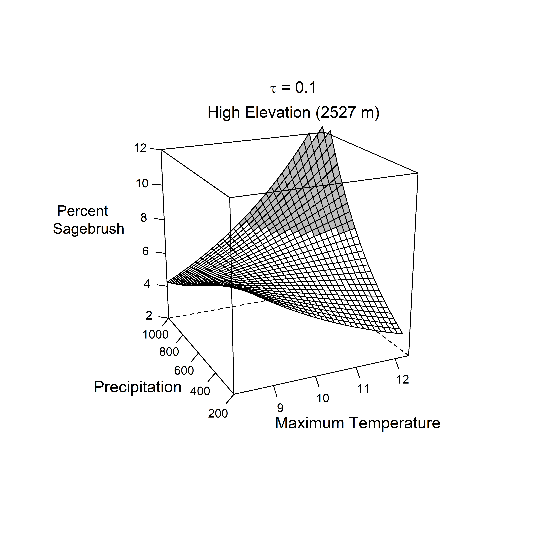


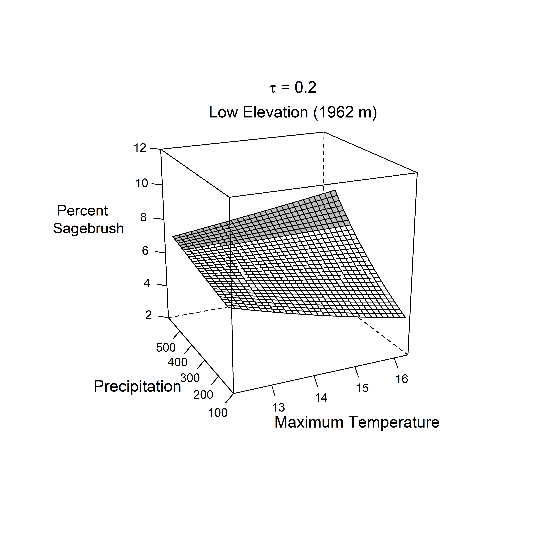

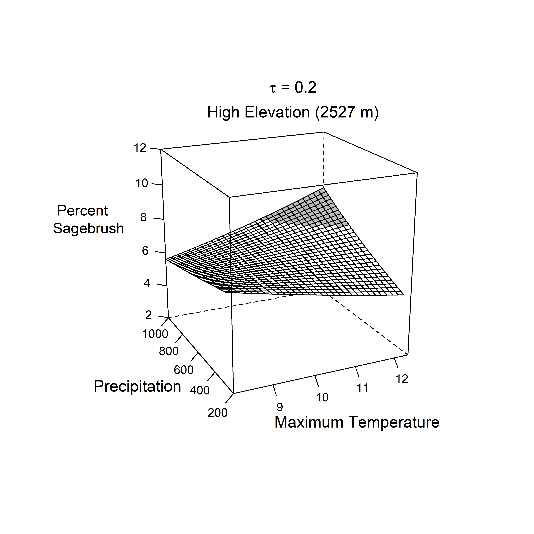


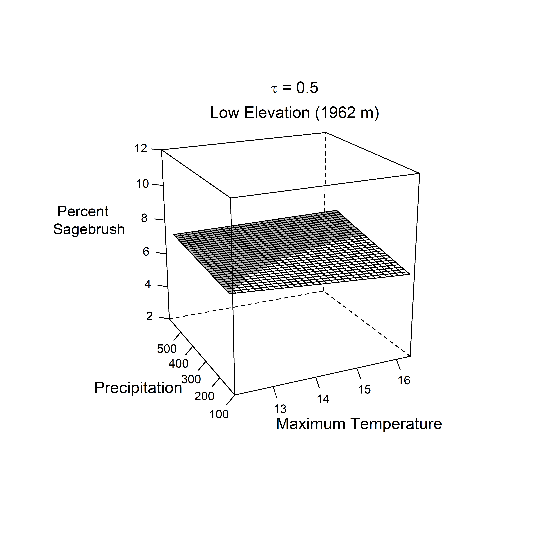

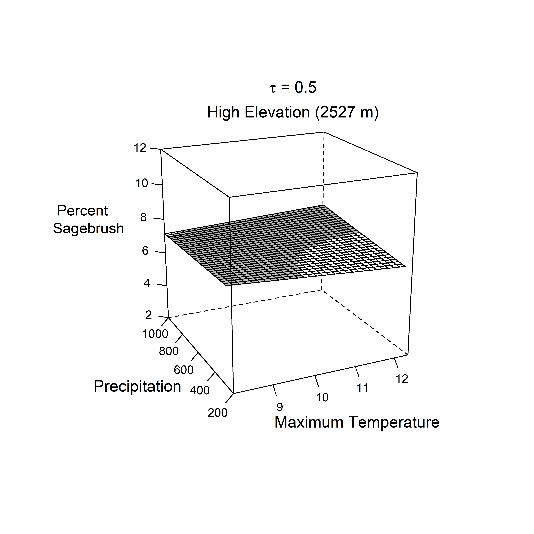


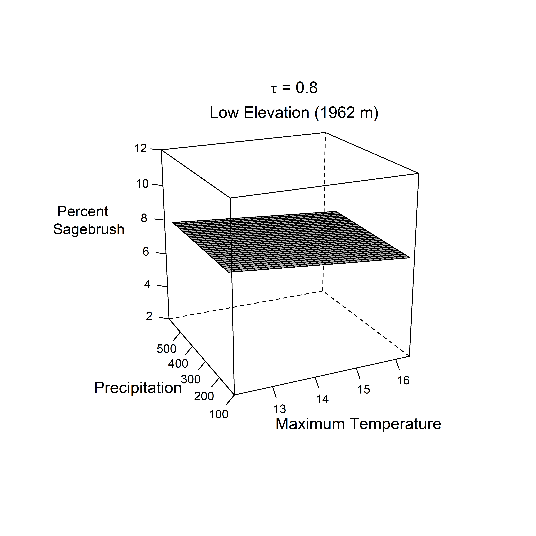

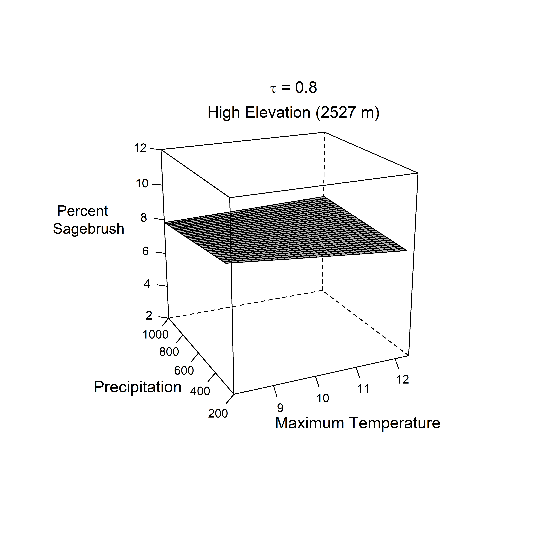


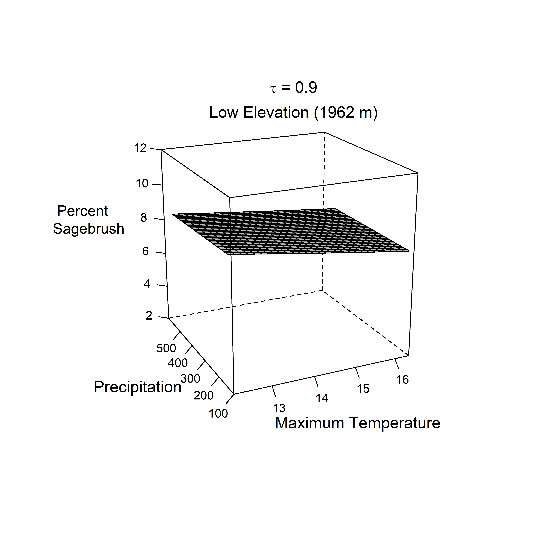

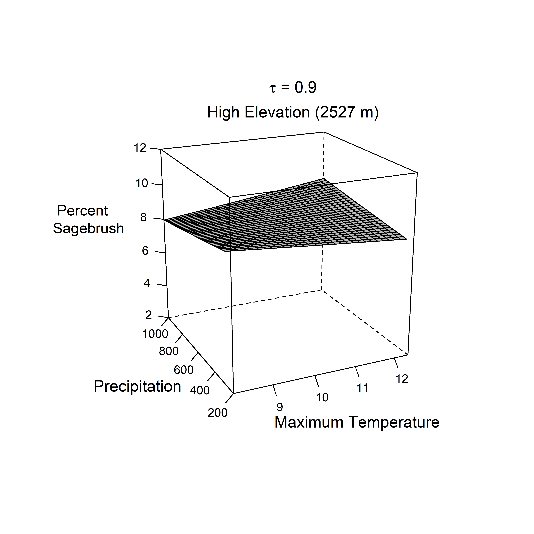


Figure S4. Predicted sagebrush (*Artemisia* spp.) cover on former oil and gas well pads in southwestern Wyoming, USA, with increasing annual precipitation and maximum temperature at low (left) and high (right) elevation and across quantiles (τ). Areas shaded gray denote an annual increase in sagebrush relative to sagebrush cover on well pads in the previous year (7.2%). We also assumed sagebrush cover on reference pixels in the current year (12.6%) based on a sample mean. The model was fit after using a general dataset to mask potential reference pixels (without local datasets on irrigated land and reservoirs, wind turbines, coal mines, and from the Density Disturbance Calculation Tool).


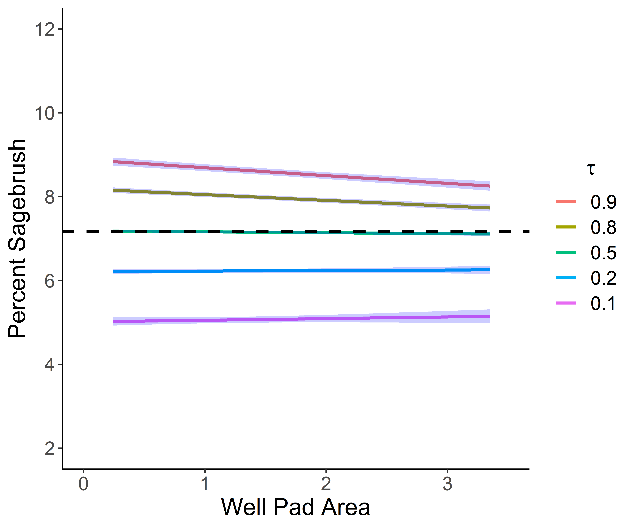

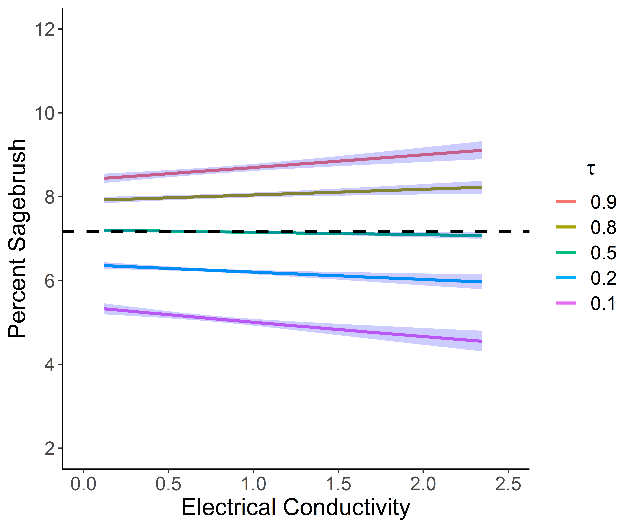

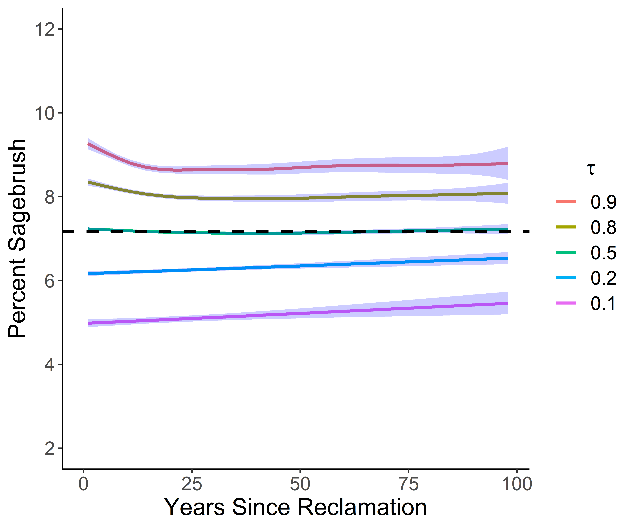

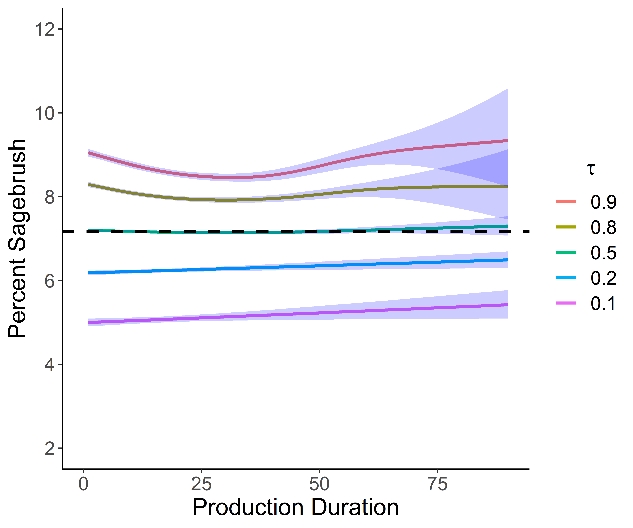

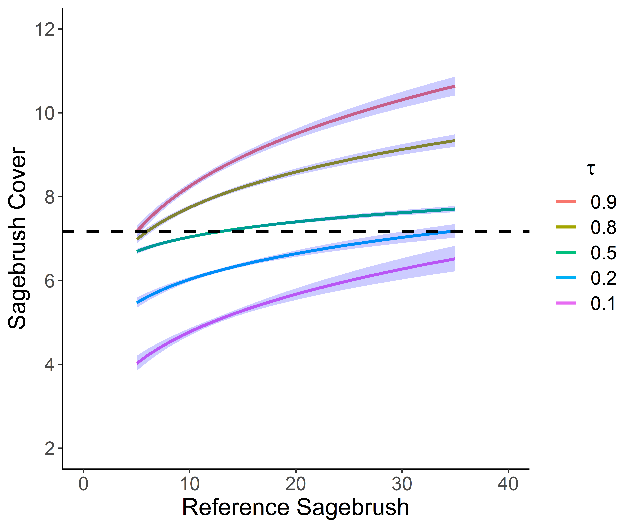


Figure S5. Predicted sagebrush (*Artemisia* spp.) cover ($\bar{x}$ ± 2 SE) on former oil and gas well pads in southwestern Wyoming, USA, with increasing well pad area (ha), electrical conductivity (dS m^–1^), years since apparent reclamation, production duration (years), and sagebrush cover (%) in reference pixels, and across quantiles (τ). For predictions, we assumed 7.2% sagebrush cover on well pads in the previous year (indicated by the horizontal dashed line). The model was fit after using a general dataset to mask potential reference pixels (without local datasets on irrigated land and reservoirs, wind turbines, coal mines, and from the Density Disturbance Calculation Tool).


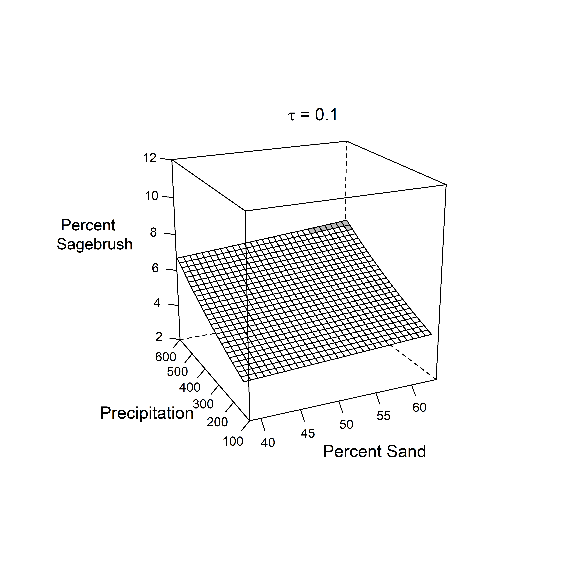

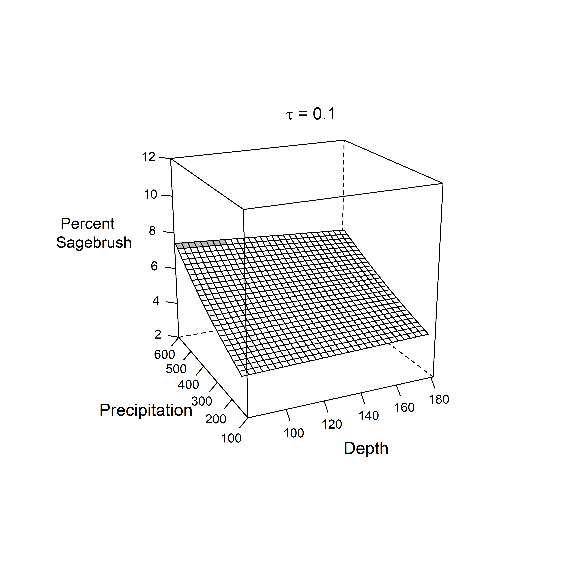


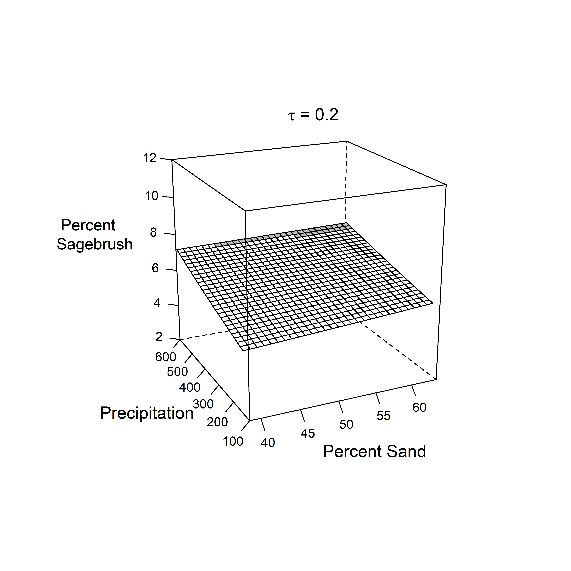

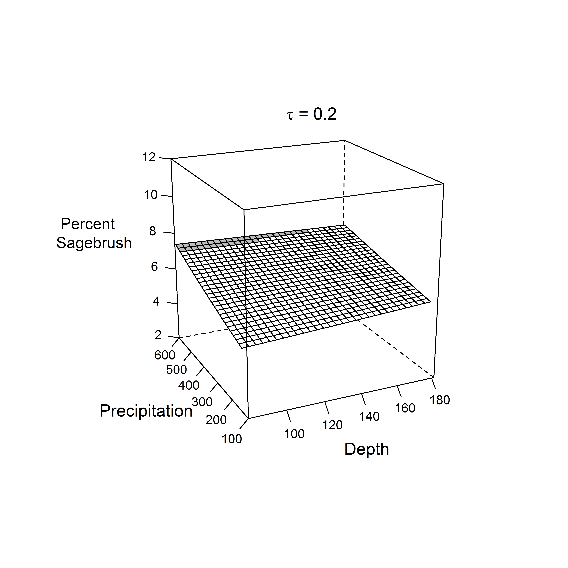


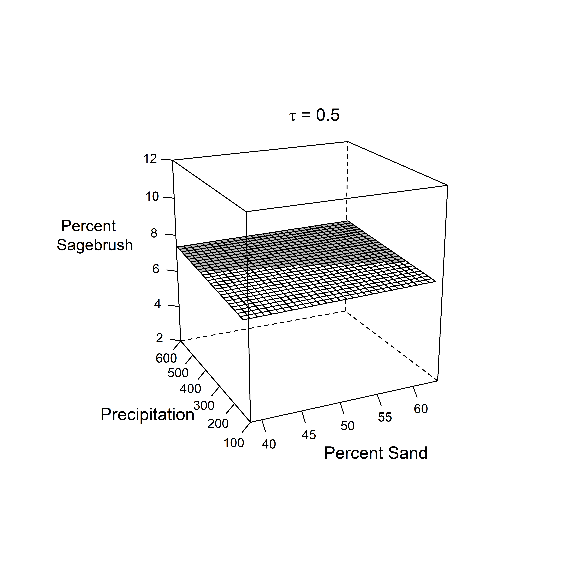

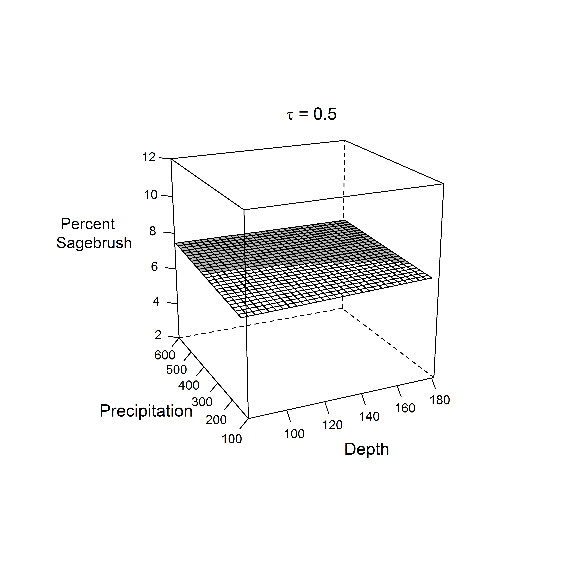


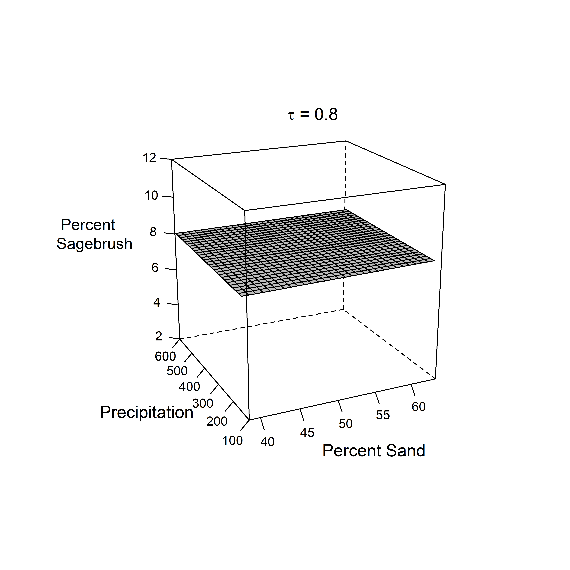

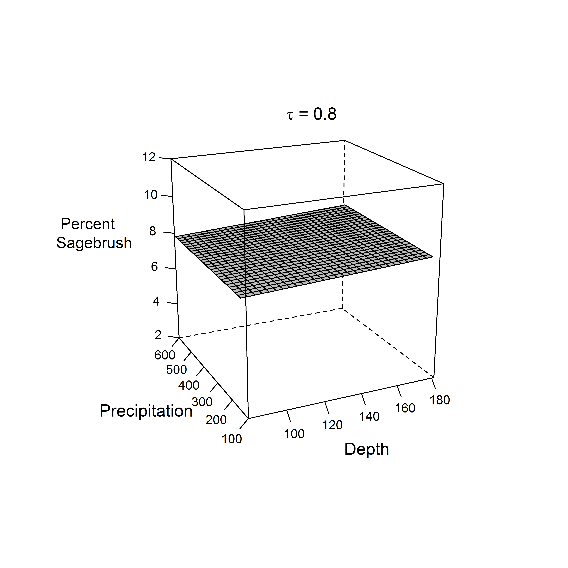


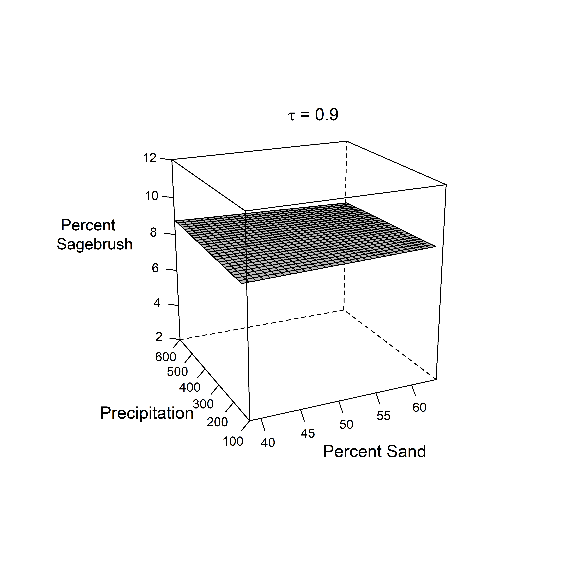

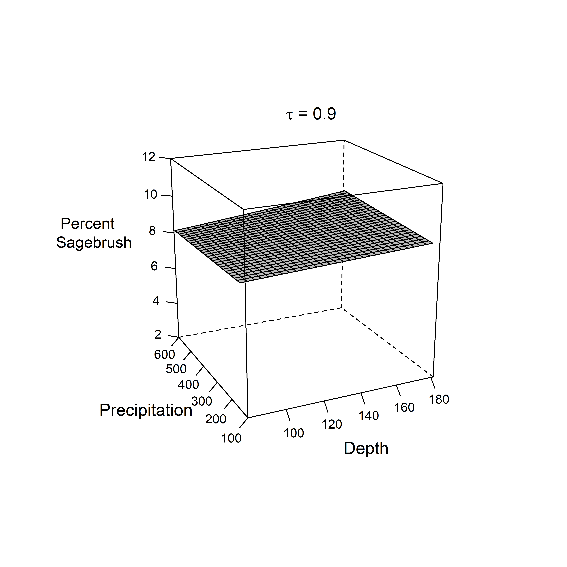


Figure S6. Predicted sagebrush (*Artemisia* spp.) cover from interactions between annual precipitation and percent sand (left) and depth to restrictive layer (cm; right) across quantiles (τ) for former oil and gas well pads in southwestern Wyoming, USA. Areas shaded gray denote an annual increase in sagebrush relative to sagebrush cover in the previous year (7.2%). We also assumed from reference pixels in the current year (12.6%) based on a sample mean. The model was fit after using a general dataset to mask potential reference pixels (without local datasets on irrigated land and reservoirs, wind turbines, coal mines, and from the Density Disturbance Calculation Tool).


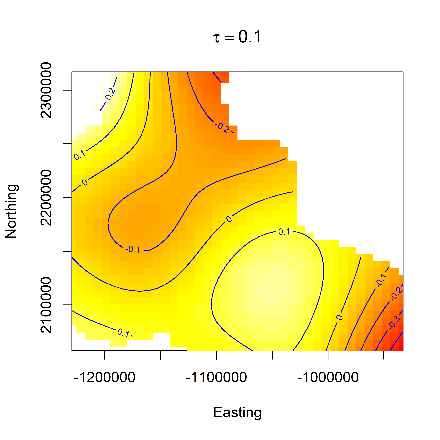

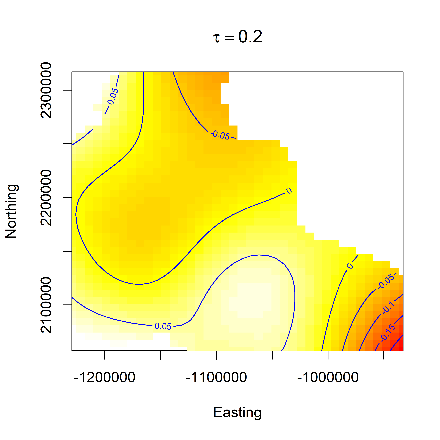

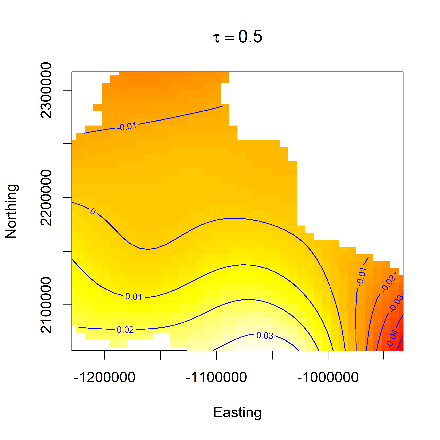

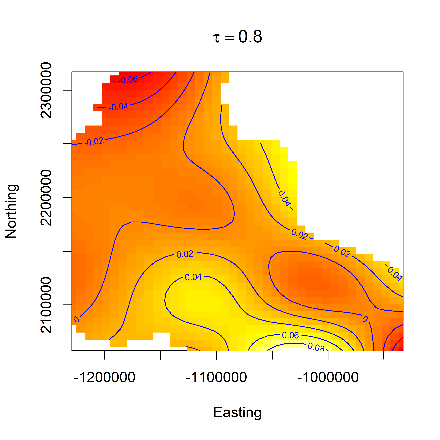

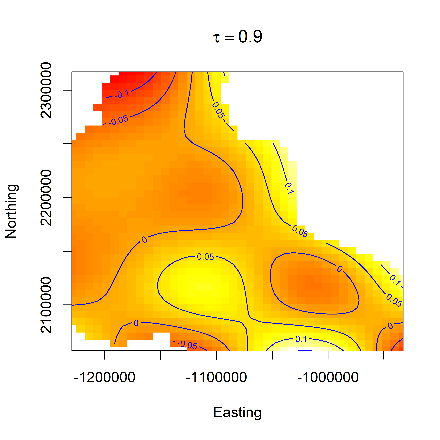


Figure S7. Heat maps for the smoothed tensor product of location (Northing and Easting) on sagebrush (*Artemisia* spp.) cover (linear predictor scale) across quantiles (τ) for former oil and gas well pads in southwestern Wyoming, USA. The model was fit after using a general dataset to mask potential reference pixels (without local datasets on irrigated land and reservoirs, wind turbines, coal mines, and from the Density Disturbance Calculation Tool). Pixel values increase from red to white.


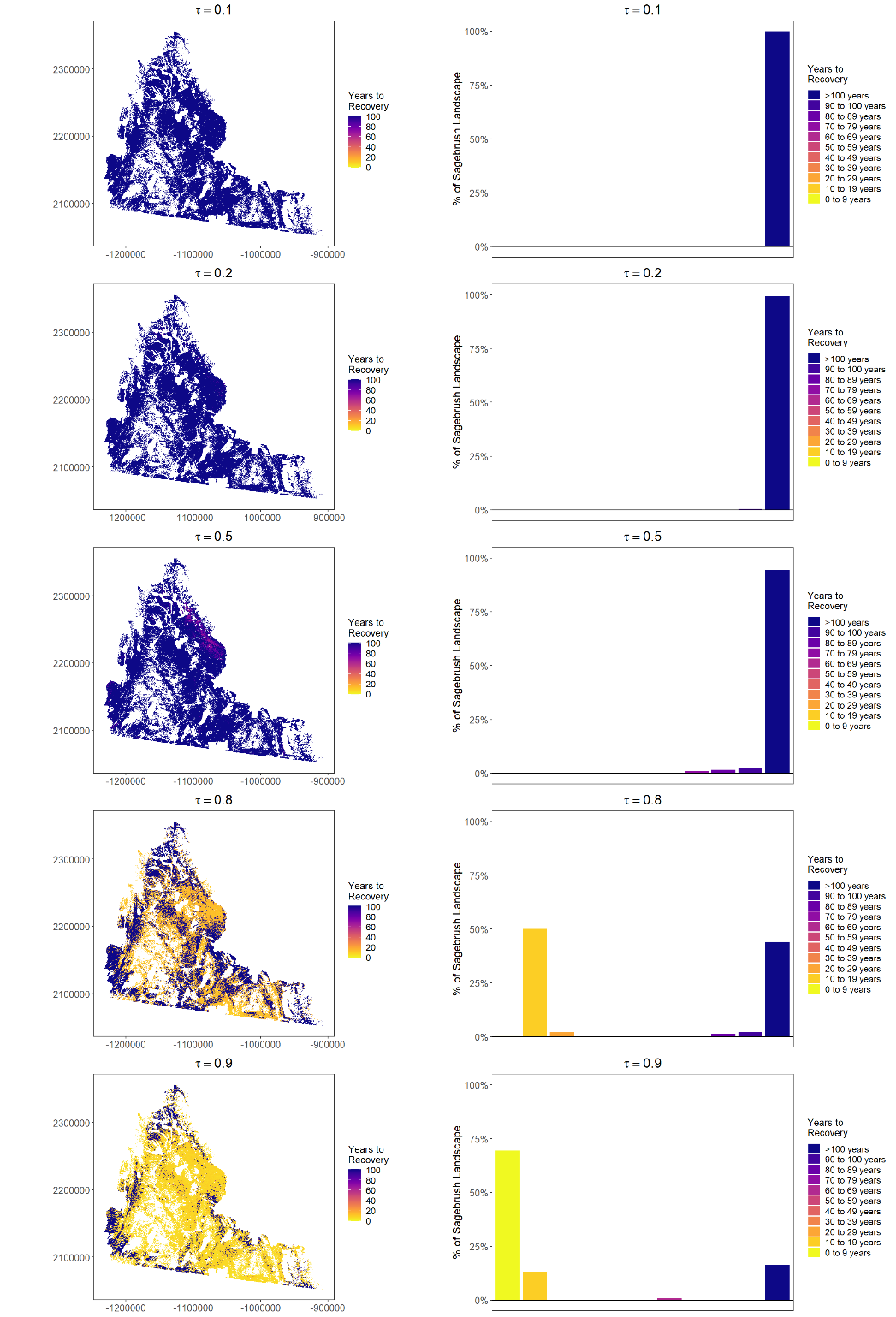


Figure S8. Projected time to recovery (years) of sagebrush (*Artemisia* spp.) cover across the study area (left) by quantile (τ) for former oil and gas well pads in southwestern Wyoming, USA. We also present histograms for each projection indicated the percentage of the sagebrush landscape grouped by years to recovery (right). The model was fit after using a general dataset to mask potential reference pixels (without local datasets on irrigated land and reservoirs, wind turbines, coal mines, and from the Density Disturbance Calculation Tool).


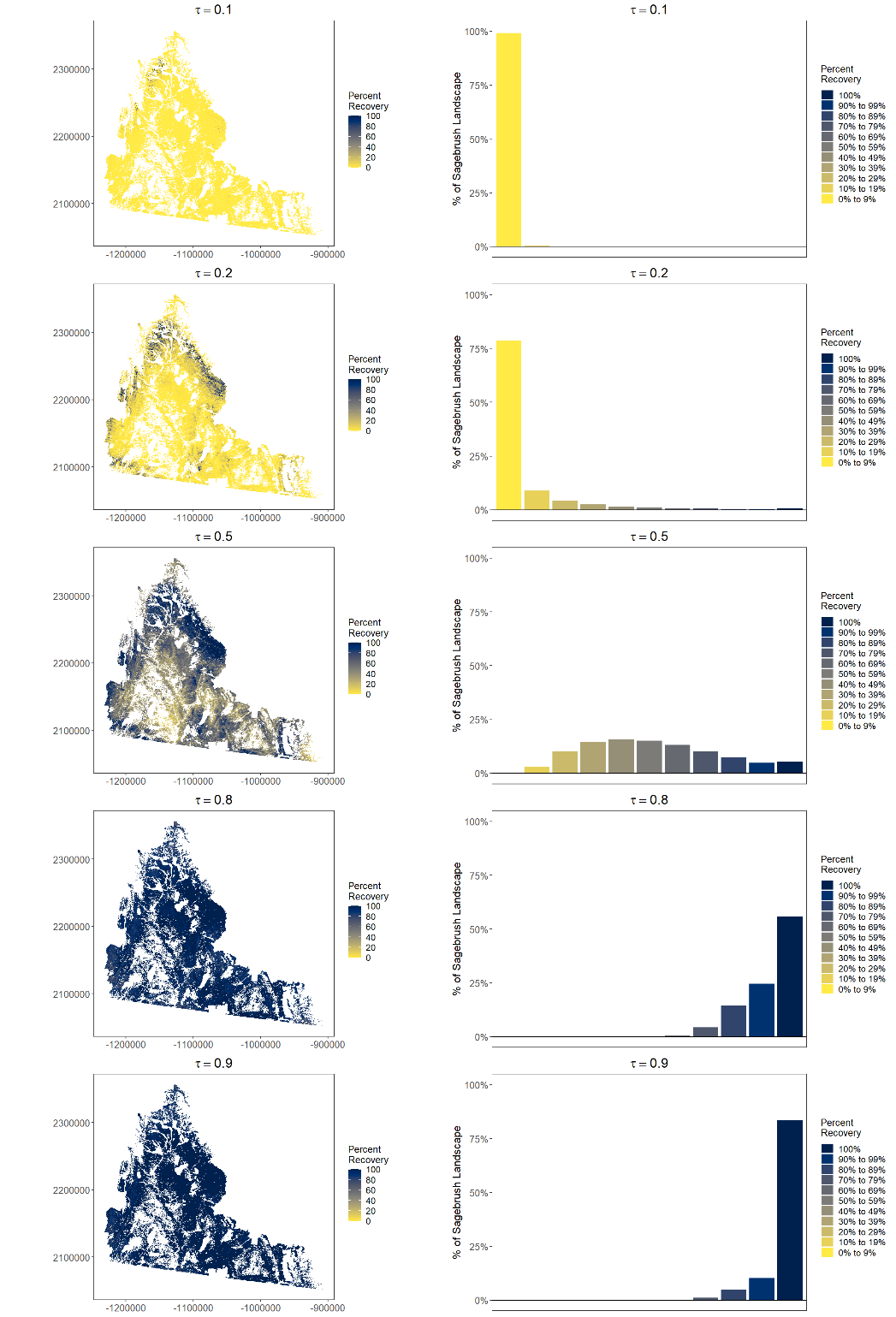


Figure S9. Projected percent recovery (relative to reference areas) of sagebrush (*Artemisia* spp.) cover across the study area (left) by quantile (τ) for former oil and gas well pads in southwestern Wyoming, USA. We also present histograms for each projection indicating the percentage of the sagebrush landscape grouped by percent recovery (right). The model was fit after using a general dataset to mask potential reference pixels (without local datasets on irrigated land and reservoirs, wind turbines, coal mines, and from the Density Disturbance Calculation Tool).


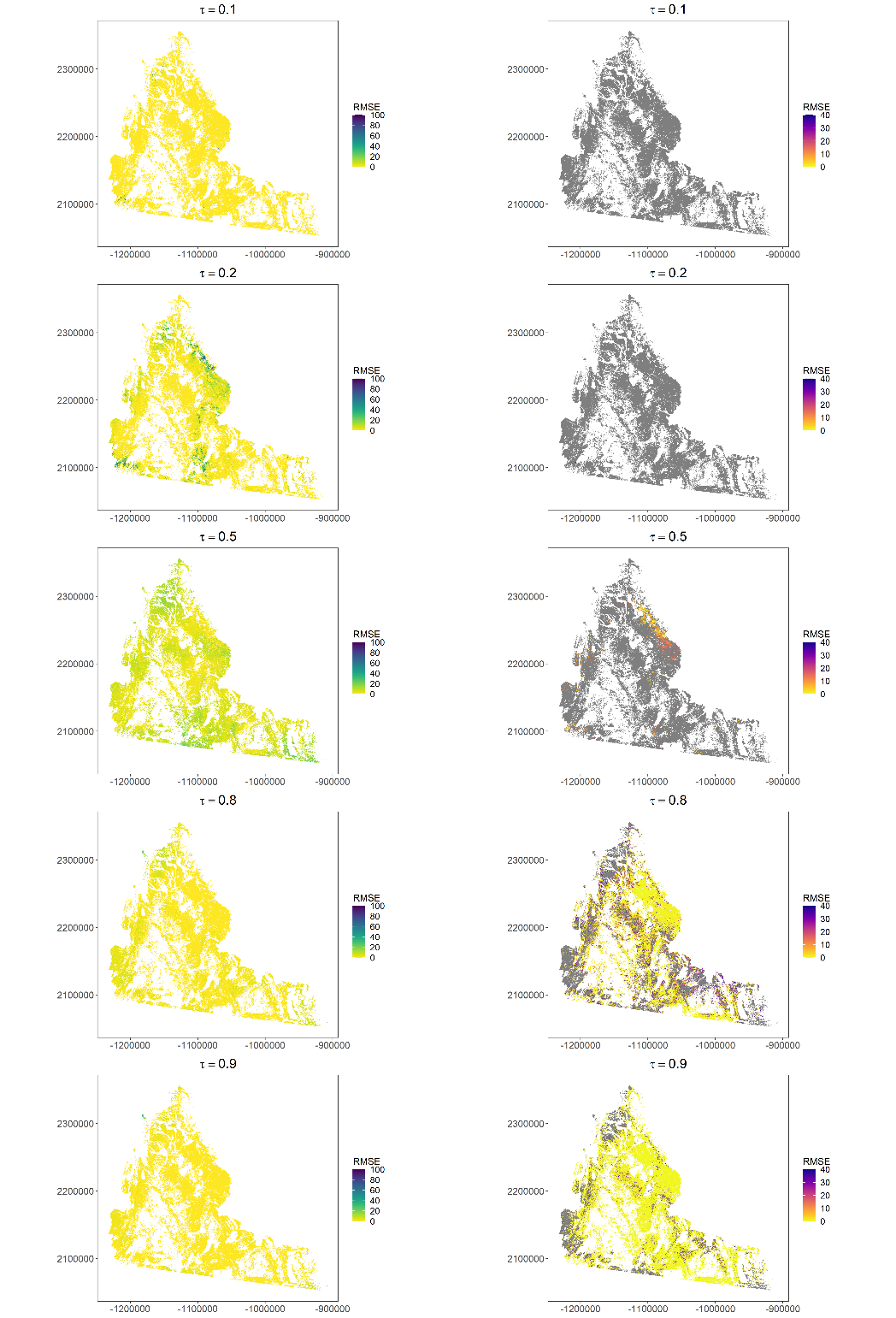


Figure S10. Root mean squared error (RMSE) for each quantile (τ) in projected percent recovery of sagebrush (*Artemisia* spp.) cover after 100 years (left) and years to recovery (right) between a model with annual weather and models with winter or spring weather. Areas in gray did not recover within 100 years based on projections from one or more models. Models were fit after using a general dataset to mask potential reference pixels (without local datasets on irrigated land and reservoirs, wind turbines, coal mines, and from the Density Disturbance Calculation Tool).


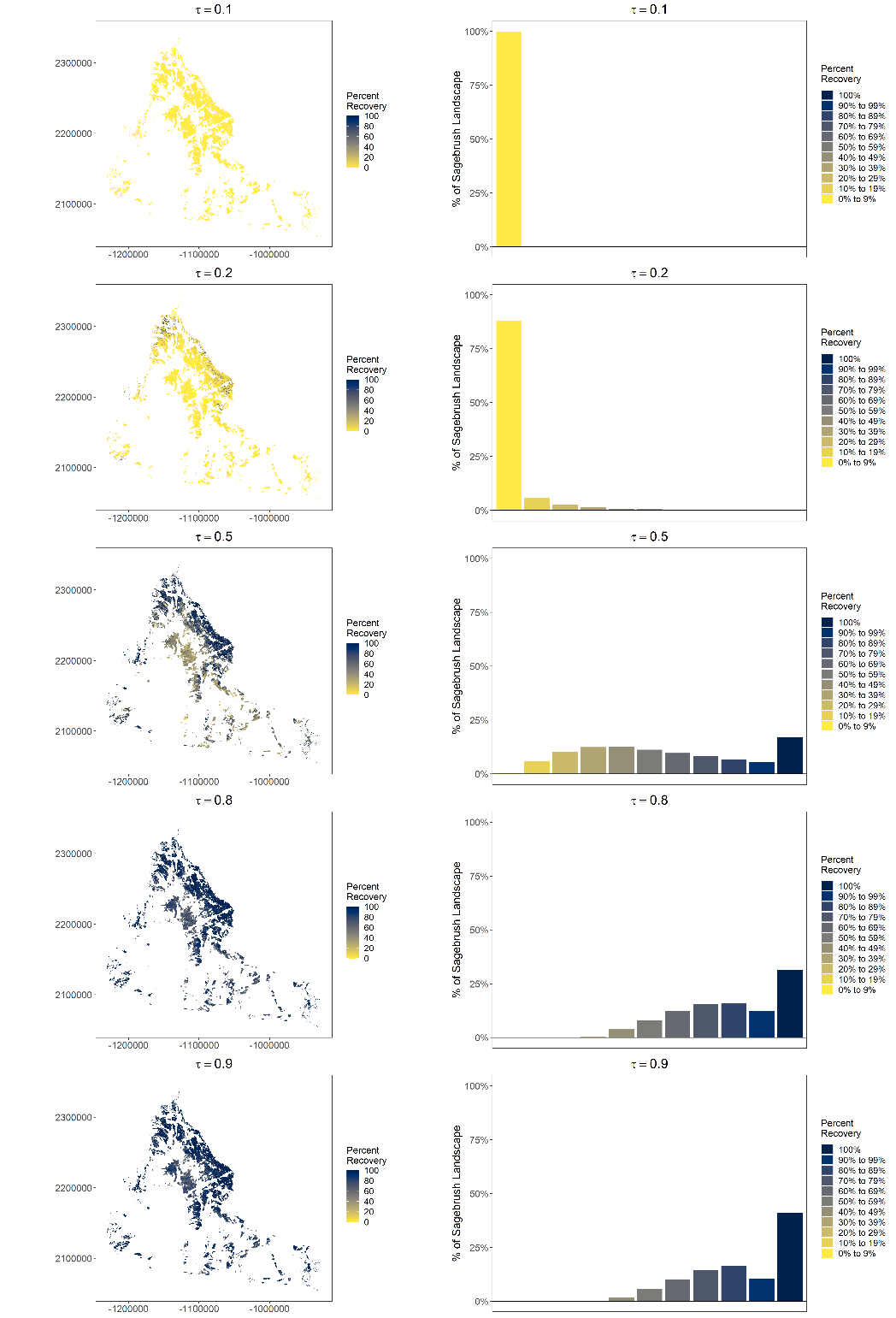


Figure S11. Projected percent recovery (relative to the 16% cover threshold) of sagebrush (*Artemisia* spp.) cover in greater sage-grouse (*Centrocercus urophasianus*) nesting habitat (left) and quantiles (τ) for former oil and gas well pads in southwestern Wyoming, USA. We also present histograms for each projection indicating the percentage of the sagebrush landscape grouped by percent recovery (right).


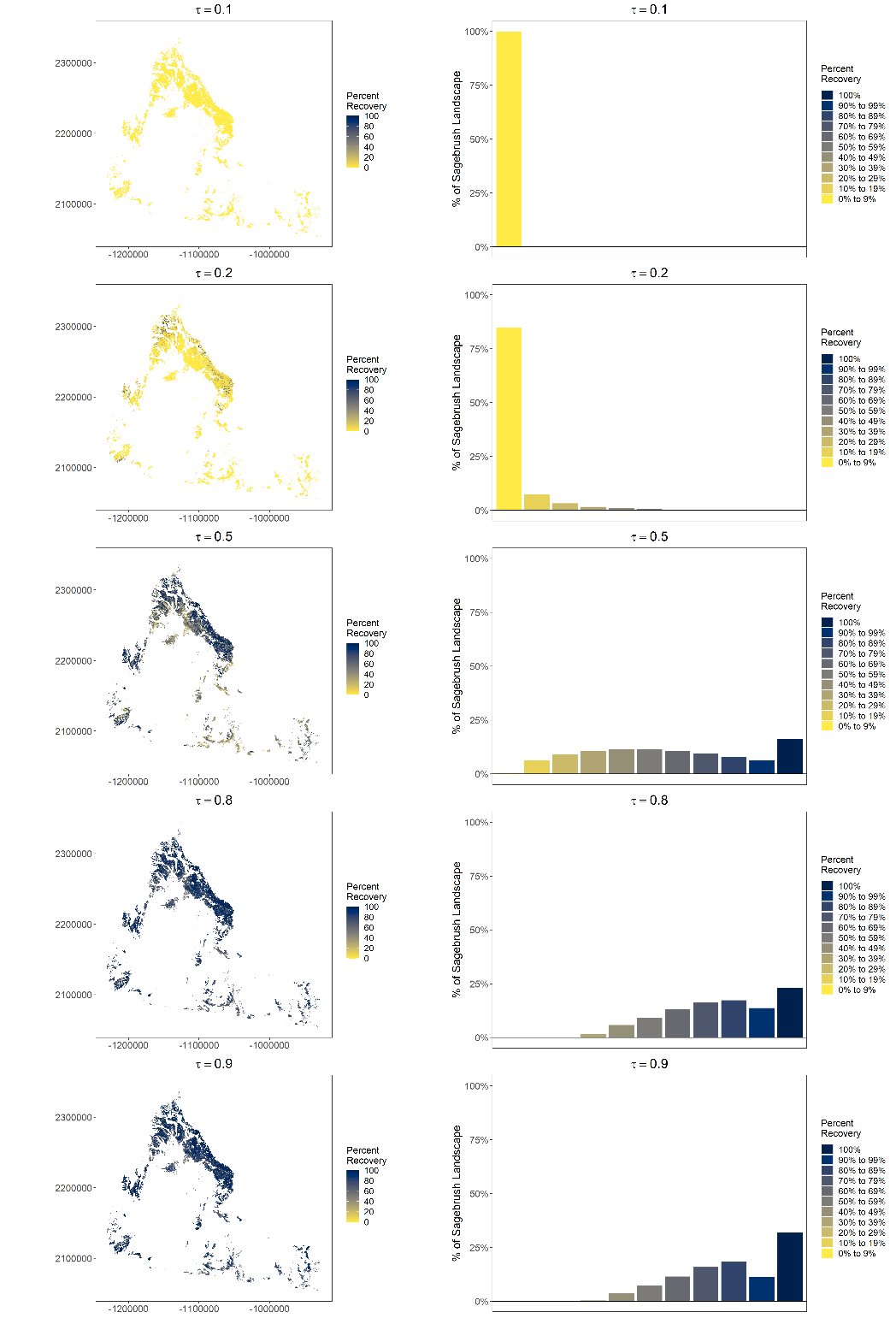


Figure S12. Projected percent recovery (relative to the 18% cover threshold) of sagebrush (*Artemisia* spp.) cover in greater sage-grouse (*Centrocercus urophasianus*) summer habitat (left) across quantiles (τ) for former oil and gas well pads in southwestern Wyoming, USA. We also present histograms for each projection indicating the percentage of the sagebrush landscape grouped by percent recovery (right).


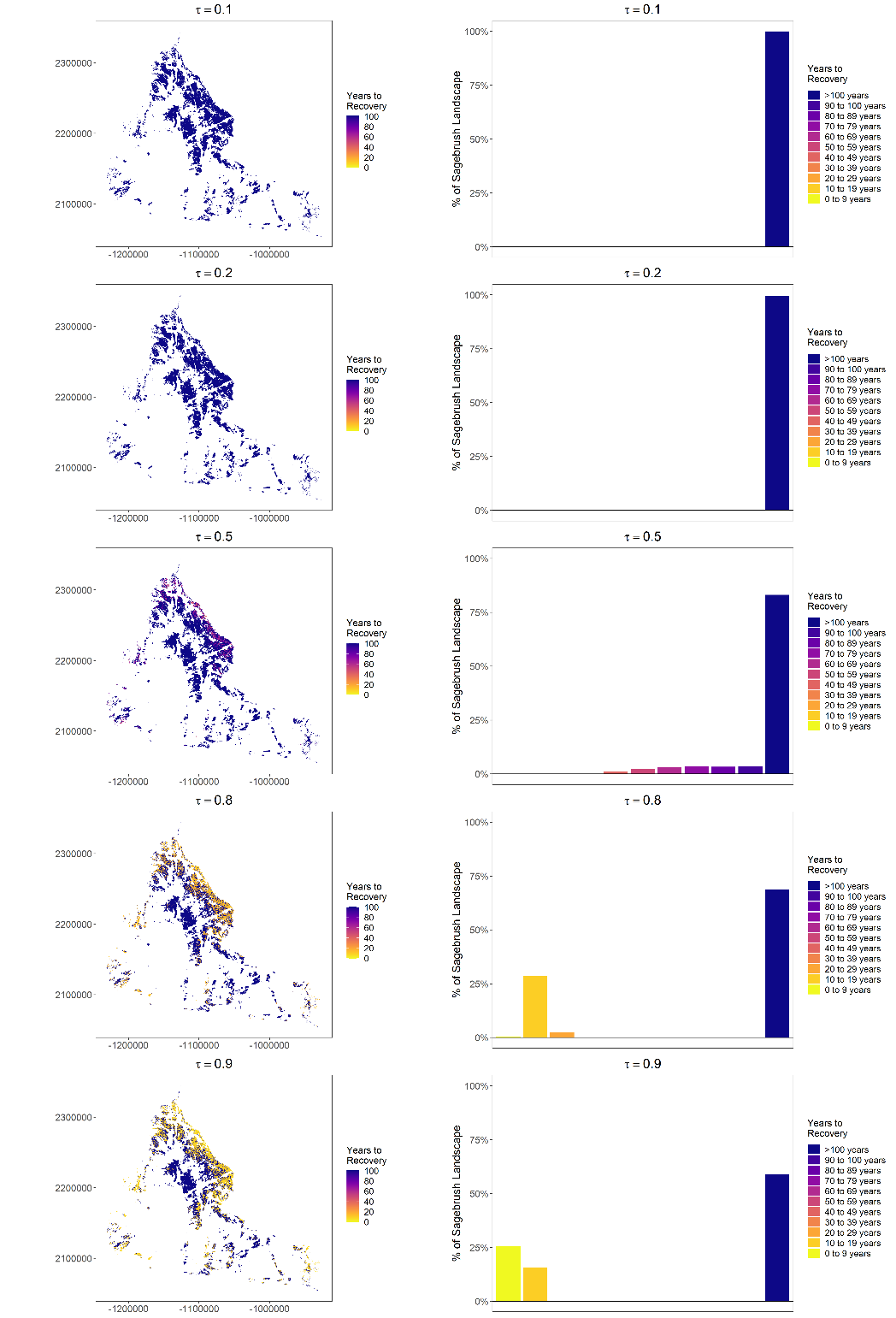


Figure S13. Projected time to recovery (years) of sagebrush (*Artemisia* spp.) cover in greater sage-grouse (*Centrocercus urophasianus*) nesting habitat (left) across quantiles (τ) for former oil and gas well pads in southwestern Wyoming, USA. We also present histograms for each projection indicating the percentage of the sagebrush landscape grouped by years to recovery (right). We assumed recovery occurred when pixels reached the threshold identified for sage-grouse nesting habitat (16% sagebrush cover).


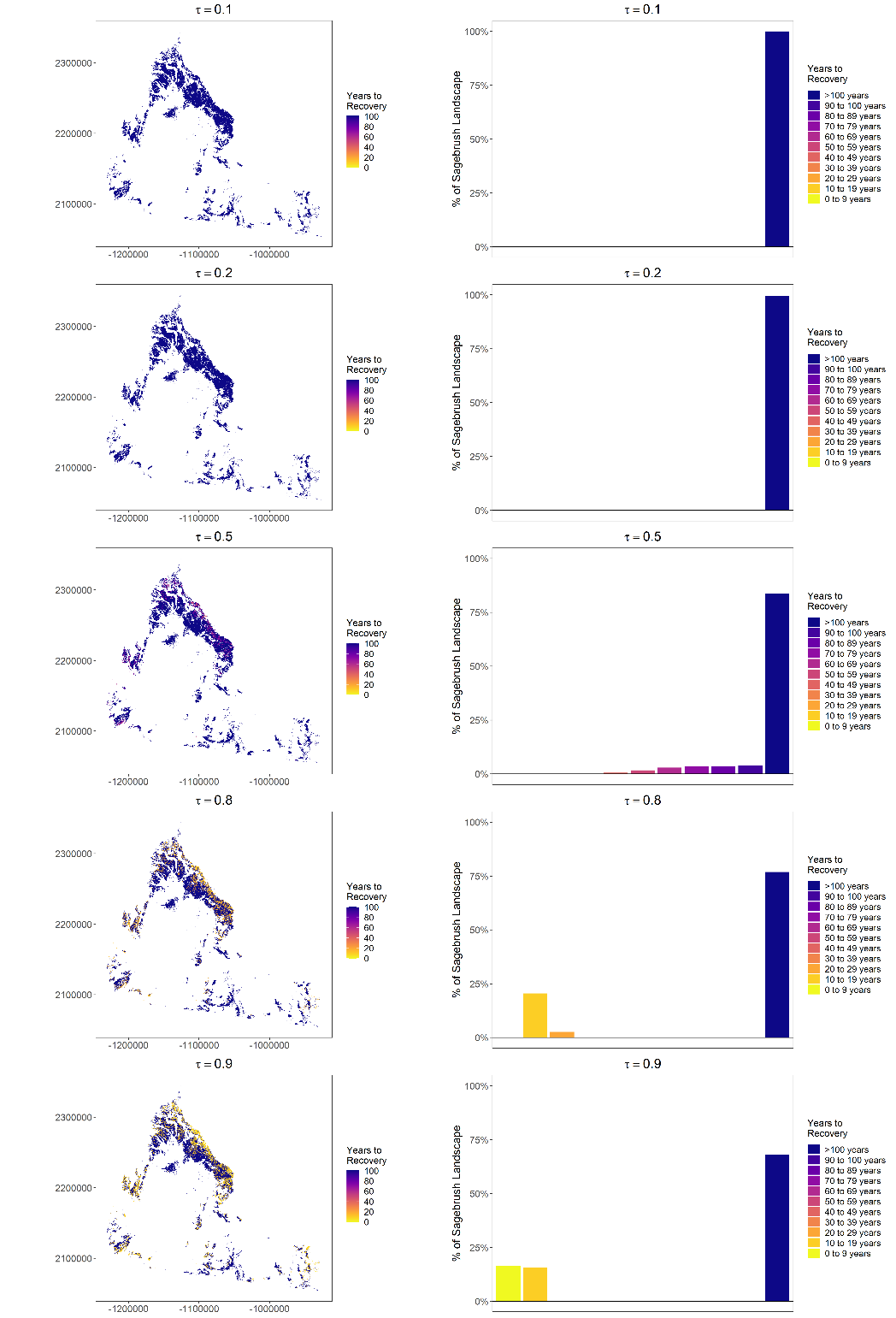


Figure S14. Projected time to recovery (years) of sagebrush (*Artemisia* spp.) cover in greater sage-grouse (*Centrocercus urophasianus*) summer (i.e., brood-rearing) habitat (left) across quantiles (τ) for former oil and gas well pads in southwestern Wyoming, USA. We also present histograms for each projection indicating the percentage of the sagebrush landscape grouped by years to recovery (right). We assumed recovery occurred when pixels reached the threshold identified for sage-grouse summer habitat (18% sagebrush cover).
